# Supplementary material for: The barley immune receptor Mla recognizes multiple pathogens and contributes to host range dynamics
Source: Nat Commun. 2021 Nov 25;12:6915. doi: 10.1038/s41467-021-27288-3 (PMC8617247; doi:10.1038/s41467-021-27288-3)
Supplement: Supplementary file 1 — Supplementary Information [file 41467_2021_27288_MOESM1_ESM.pdf]

The barley immune receptor *Mla* recognizes multiple pathogens and contributes to host range dynamics

Bettgenhaeuser *et al*.

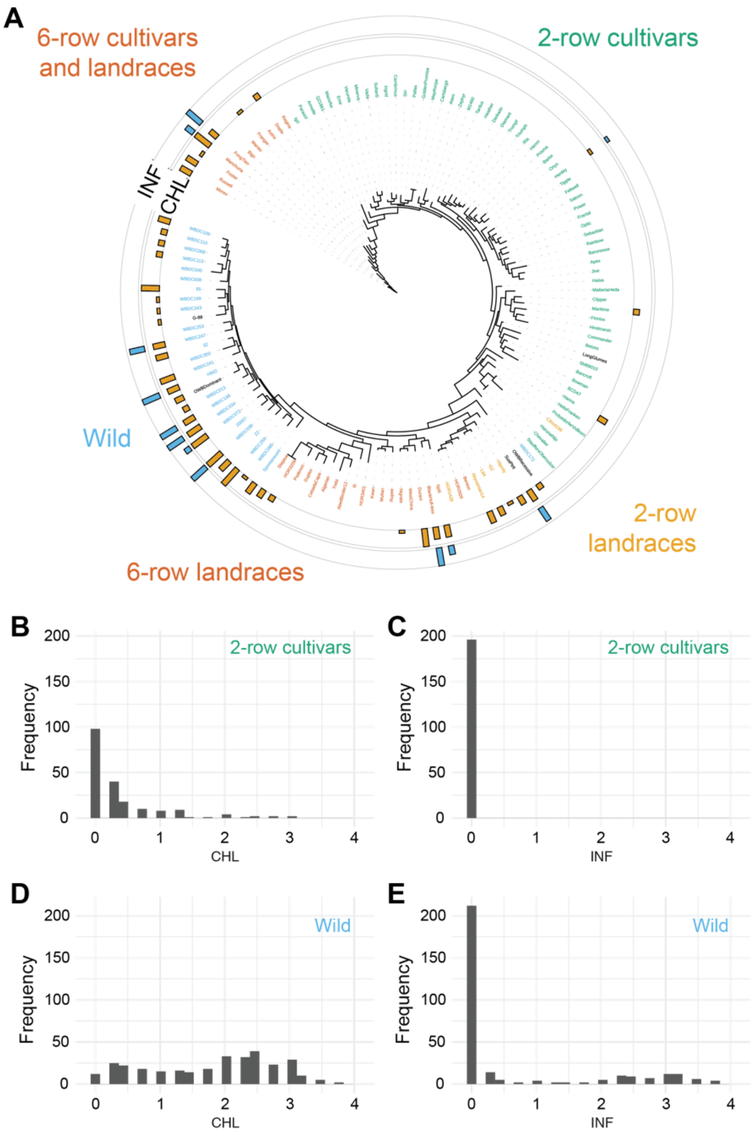

Supplementary Figure 1. Unequal distribution of resistance to *Puccinia striiformis* f. sp. *tritici* isolate 08/21 in diverse barley germplasm. (A) A panel of 129 barley accessions was genotyped using the barley OPA1 platform[1]. 1,258 SNPs were used to generate a dendrogram using maximum likelihood. Four clades were classified based on agronomy (cultivar, landrace, and wild) and row-status (two-row or six-row) of accessions. Accessions in black are genetic stocks or have uncertain status. Macroscopic phenotypes using 0 to 4 scales in 0.5 increments are displayed in surrounding rings, with inner and outer rings displaying chlorosis (hyphal colonization) and infection (pustule formation), respectively. (B) and (C) 196 domesticated, 2-row spring-type elite barley cultivars from Europe (AGOUEB panel[2]) were inoculated with *P. striiformis* f. sp. *tritici* isolate 08/21 and assessed for chlorosis and infection, respectively. (D) and (E) A collection of 313 wild barley (*H. vulgare* subsp. *spontaneum*) accessions from the WBDC panel[3] were inoculated with *P. striiformis* f. sp. *tritici* isolate 08/21 and assessed for (D) chlorosis and (E) infection. In the AGEOUB panel, no accessions showed pustule formation, whereas 101 accessions (32%) in the WBDC panel showed some degree of pustule formation. Two independent replicates were performed. Data shown is the average of those replicates. Source data are provided as a Source Data file.

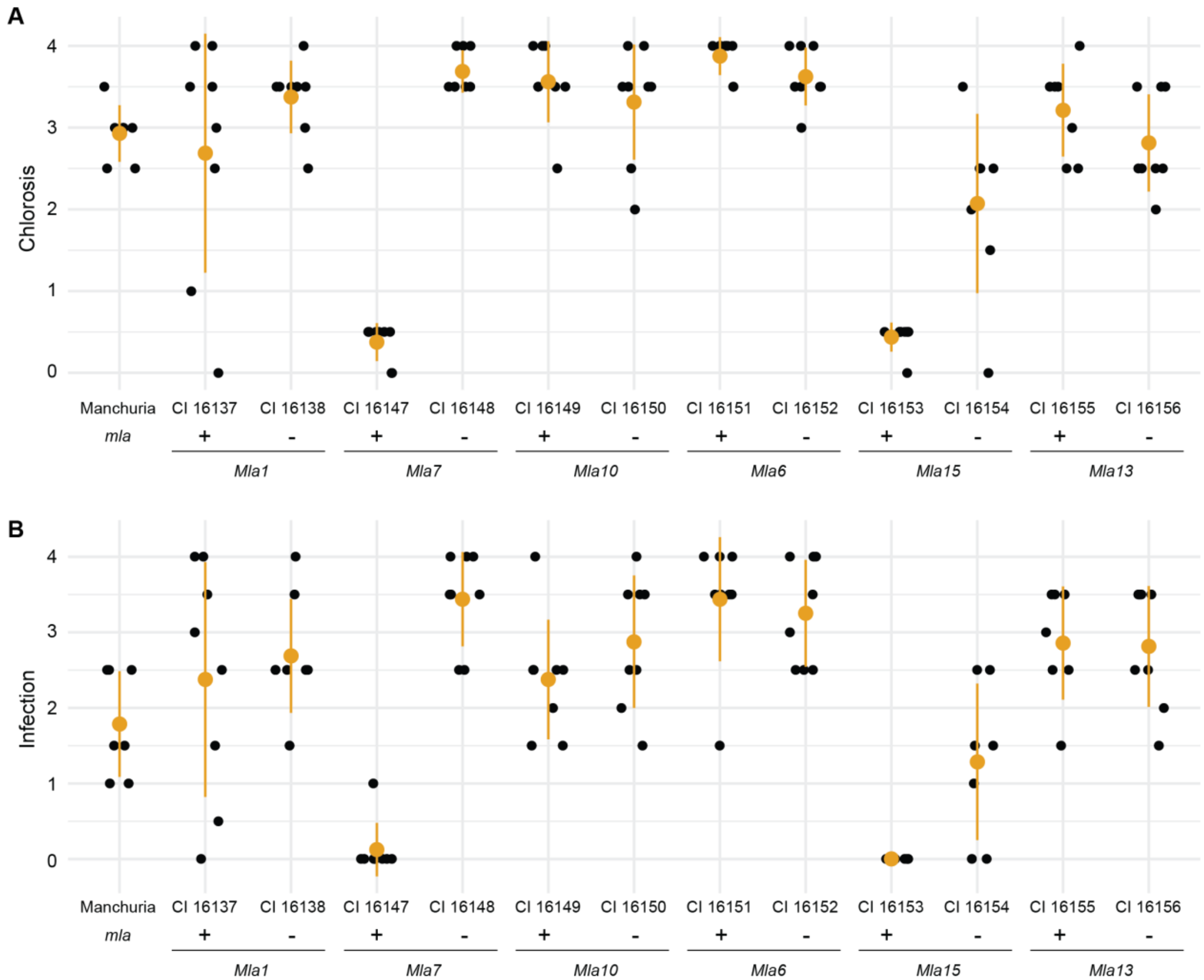

Supplementary Figure 2. Coupling of *Mla7* and *Mla15* with *Rps7* in the Manchuria near-isogenic lines. The Manchuria near-isogenic lines[4] were inoculated with *Pst* isolate 08/21 and phenotyped for (A) chlorosis and (B) infection at 14 days post inoculation. Phenotypes range from 0 to 4 with increments of 0.5. Paired isogenic lines contain an *Mla* allele or the Manchuria allele (*mla*), which encodes an *RGH1* pseudogene. Mean (dot) and standard deviation (line) are shown in yellow and individual data points in black based on eight independent first leaves for each barley accession (N=8). Source data are provided as a Source Data file.

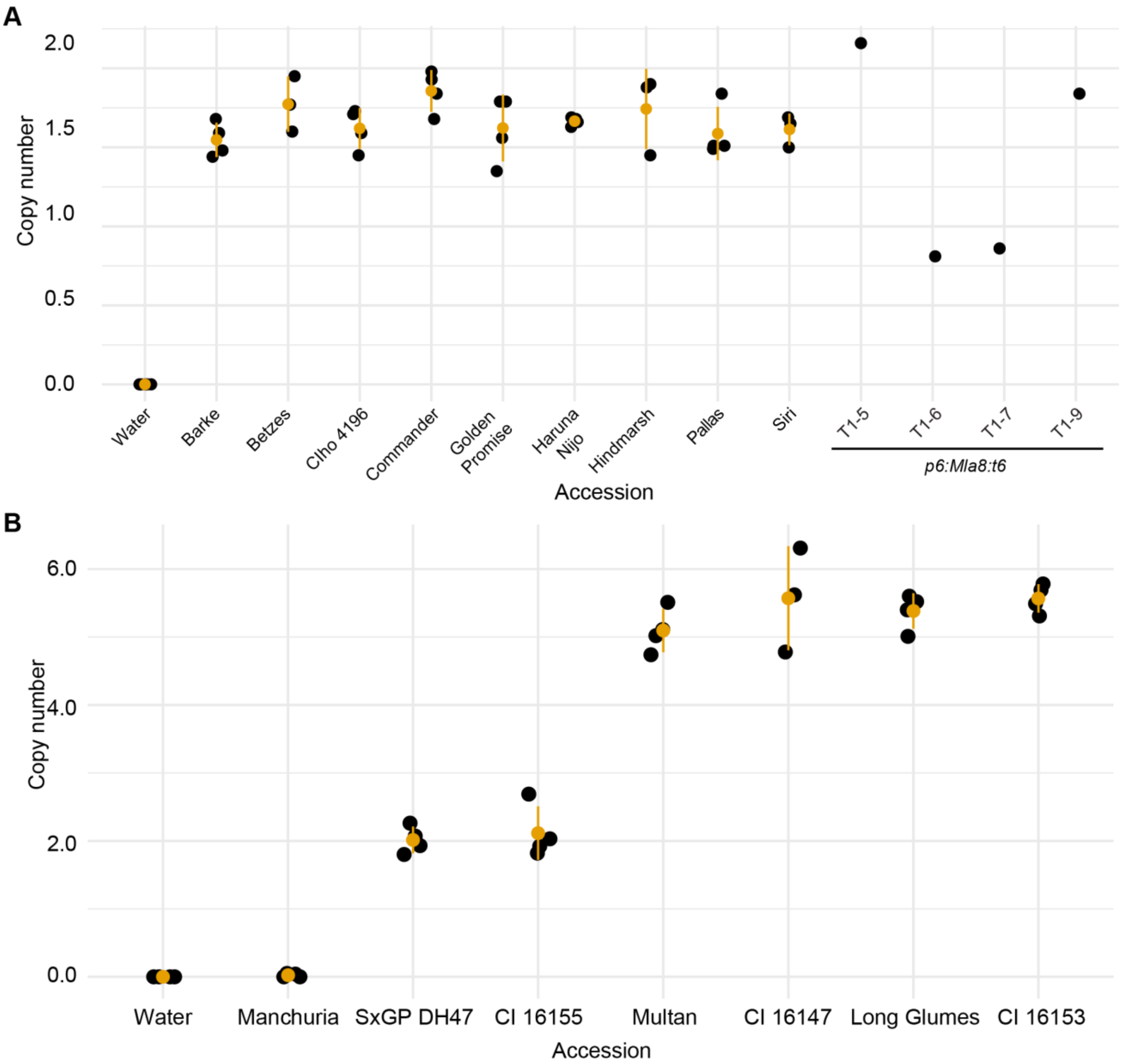

Supplementary Figure 3. Copy number variation in *Mla8* and *Mla7*. (A) Digital droplet PCR with primers developed on *Mla8* was performed on barley (diploid, homozygous) accessions carrying *Mla8* (Barke, Betzes, CIho 4196, Commander, Golden Promise, Haruna Nijo, Hindmarsh, Pallas, and Siri), transgenic SxGP DH-47 lines carrying *p6:Mla8:p6* construct (T1-5, T1-6, T1-7, and T1-9), and water control. (B) Digital droplet PCR with primers developed on *Mla7* was performed on accessions carrying *Mla7* (Multan and CI 16147) and *Mla15* (Long Glumes and CI 16153). Controls include water, Manchuria (*RGH1bcd*), SxGP DH-47 (*RGH1.SusPtrit*), and CI 16155 (*Mla13*). The PCR primers amplify from a region that is conserved between *Mla7*, *Mla13*, and *RGH1.SusPtrit*, but not conserved with *RGH1bcd*. Mean (dot) and standard deviation (line) are shown in yellow and individual data points in black. Experiments were performed with either three or four replicates using independently sampled and extracted genomic DNA (N >= 3), except for primary transgenic lines which have no replication (N=1). Source data are provided as a Source Data file.

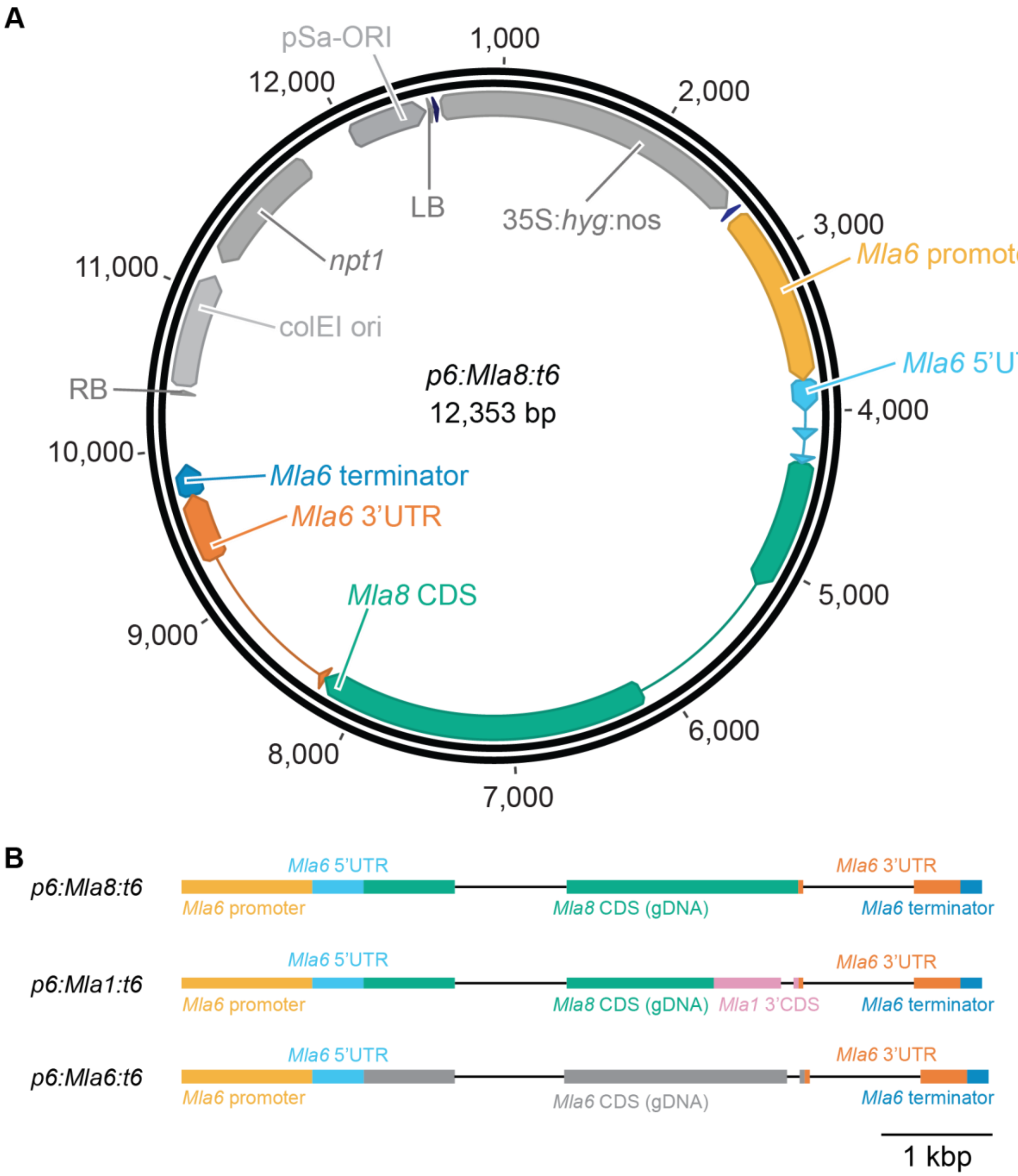

Supplementary Figure 4. Plasmid map and construct design for plant transformation of diverse *Mla* alleles. (A) Plasmid map of the genomic fragment encompassing the coding sequence of *Mla8* inserted in the promoter-5'UTR and 3'UTR-terminator of *Mla6* (*p6:Mla8:t6*) construct used for plant transformation. (B) Design of expression constructs for expression of *Mla1*, *Mla6*, and *Mla8*. Sequences are available on NCBI GenBank accessions MZ555767 (*p6:Mla8:t6*), MZ555768 (*p6:Mla1:t6*), MZ555769 (*p6:Mla6:t6*). Source data are provided as a Source Data file.

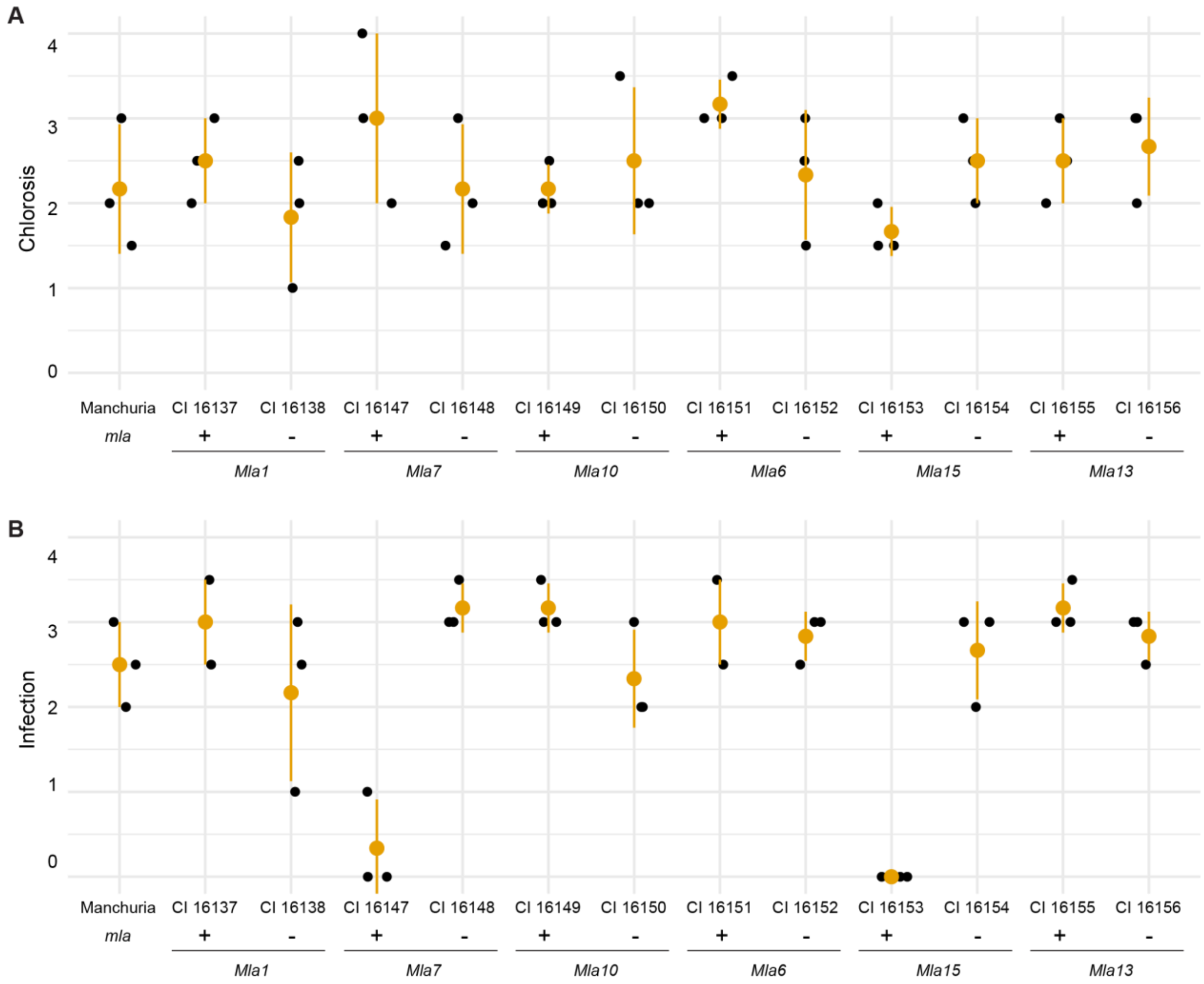

Supplementary Figure 5. Barley accessions CI 16147 (*Mla7*) and CI 16153 (*Mla15*) are resistant to *P. striiformis* f. sp. *tritici* isolate 16/035. The Manchuria near-isogenic lines[4] were inoculated with *Pst* isolate 16/035 and phenotyped for (A) chlorosis and (B) infection at 14 days post inoculation. Phenotypes range from 0 to 4 with increments of 0.5. Paired isogenic lines contain an *Mla* allele or the Manchuria allele (*mla*), which encodes an *RGH1* pseudogene. Mean (dot) and standard deviation (line) are shown in yellow and individual data points in black based on three independent first leaves for each barley accession (N=3). Source data are provided as a Source Data file.

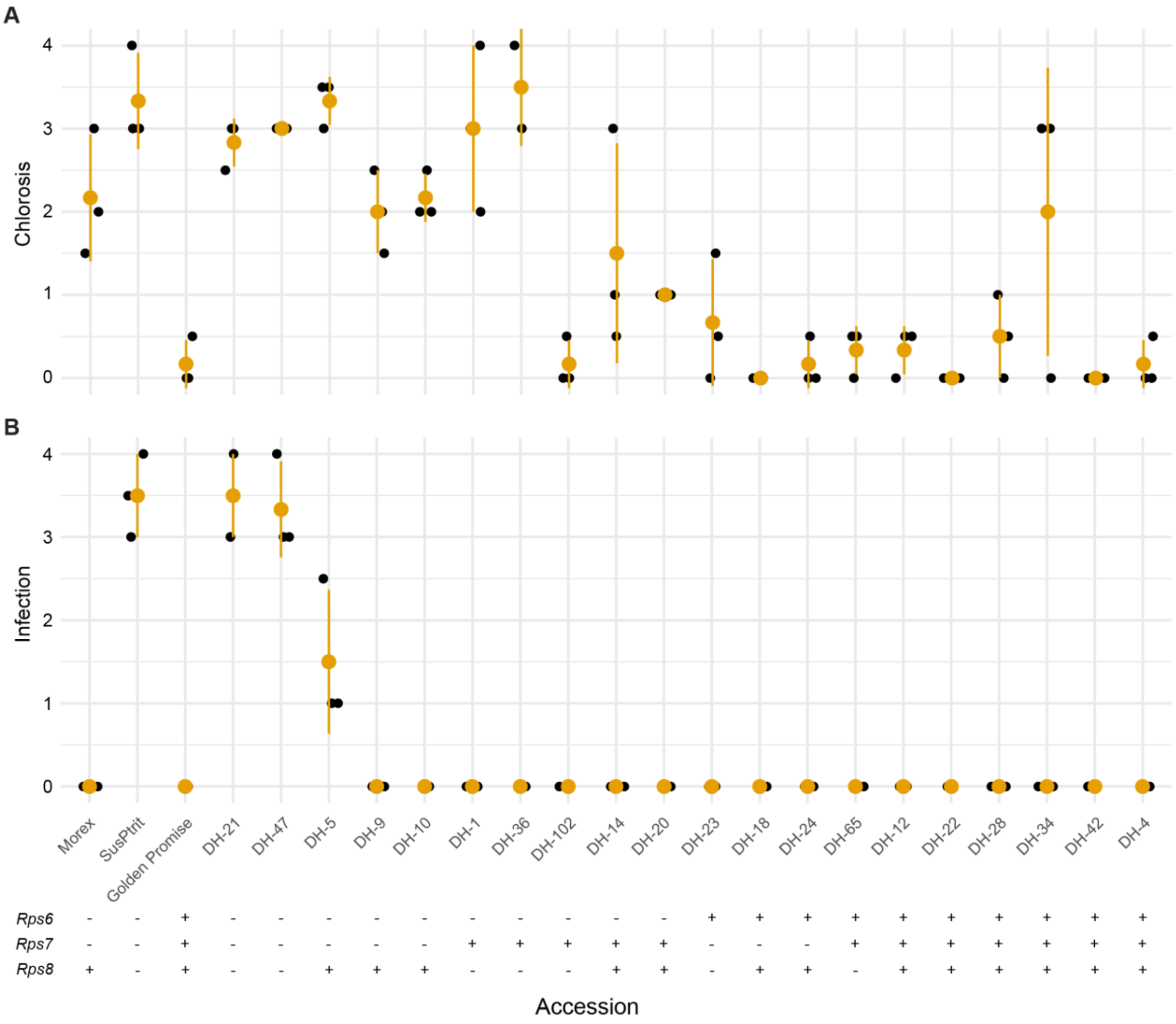

Supplementary Figure 6. Segregation for resistance to *P. striiformis* f. sp. *tritici* isolate 16/035 in a subset of the SxGP DH population. Phenotype x genotype plots for (A) chlorosis and (B) infection show the contribution of *Rps6*, *Rps7*, and *Rps8* to resistance to *P. striiformis* f. sp. *tritici* isolate 16/035 in a subset of accessions in the SusPtrit x Golden Promise doubled haploid population. Presence (+) or absence (-) of *Rps6*, *Rps7*, and *Rps8* for each DH line are indicated in the bottom of the figure based on the markers SCRI_RS_155652, BOPA2_12_30817, and BOPA1_4361−1867, respectively. Mean (dot) and standard deviation (line) are shown in yellow and individual data points in black based on three independent first leaves for each barley accession (N=3). Source data are provided as a Source Data file.

Supplementary references

1 Close, T. J. *et al.* Development and implementation of high-throughput SNP genotyping in barley. *BMC Genomics* **10**, 582 (2009).

2 Thomas, W. *et al.* HGCA Project Report 528: Association genetics of UK elite barley (AGOUEB). (Agriculture and Horticulture Development Board, 2014).

3 Steffenson, B. J. *et al.* A walk on the wild side: mining wild wheat and barley collections for rust resistance genes. *Aust. J. Agric. Res.* **58**, 532-544 (2007).

4 Moseman, J. G. Isogenic barley lines for reaction to *Erysiphe graminis* f. sp. *hordei*. *Crop Sci.* **12**, 681-682 (1972).
