# Supplementary material for: The barley immune receptor Mla recognizes multiple pathogens and contributes to host range dynamics
Source: Nat Commun. 2021 Nov 25;12:6915. doi: 10.1038/s41467-021-27288-3 (PMC8617247; doi:10.1038/s41467-021-27288-3)
Supplement: Supplementary file 3 — Description of Additional Supplementary Files [file 41467_2021_27288_MOESM3_ESM.pdf]

Description of Additional Supplementary Files

File Name: Supplementary Data 1
Description: Origin and descriptive information for barley germplasm used in this study.

File Name: Supplementary Data 2
Description: Composite interval mapping of resistance to P. striiformis f. sp. tritici in doubled-haploid and recombinant inbred line mapping populations.

File Name: Supplementary Data 3
Description: Composite interval mapping of resistance to P. striiformis f. sp. tritici in four F2 mapping populations.

File Name: Supplementary Data 4
Description: Marker-trait association at the Rps6, Rps7, and Rps8 loci in diverse barley F2 and BC1 populations inoculated with P. striiformis f. sp. tritici.

File Name: Supplementary Data 5
Description: Structured populations and near isogenic lines inoculated with P. striiformis f. sp. tritici.

File Name: Supplementary Data 6
Description: Phenotypic evaluation of barley carrying Mla1, Mla6, Mla7, and Mla8 transgenes with diverse B. graminis f. sp. hordei isolates.

File Name: Supplementary Data 7
Description: Mla haplotypes associated with Rps7-mediated resistance.

File Name: Supplementary Data 8
Description: CAPS and SSLP markers used for genetic map construction, linkage analysis of Rps6, Rps7, and Rps8, and recombination screens and fine-mapping Rps7.

File Name: Supplementary Data 9
Description: KASP markers used for genetic map construction, linkage analysis of Rps6, Rps7, and Rps8, and fine-mapping Rps7.

File Name: Supplementary Data 10
Description: Sequenom markers used for genetic map construction.

File Name: Supplementary Data 11
Description: Primer and synthesized sequences used for Gibson cloning of T-DNA constructs carrying Mla alleles driven by the Mla6 promoter.
