# Supplementary material for: The barley immune receptor Mla recognizes multiple pathogens and contributes to host range dynamics
Source: Nat Commun. 2021 Nov 25;12:6915. doi: 10.1038/s41467-021-27288-3 (PMC8617247; doi:10.1038/s41467-021-27288-3)
Supplement: Supplementary file 15 — Reporting Summary [file 41467_2021_27288_MOESM15_ESM.pdf]

nature portfolio

Corresponding author(s): Matthew Moscou
Last updated by author(s): Nov 5, 2021

Reporting Summary

Nature Portfolio wishes to improve the reproducibility of the work that we publish. This form provides structure for consistency and transparency in reporting. For further information on Nature Portfolio policies, see our Editorial Policies and the Editorial Policy Checklist.

Statistics

For all statistical analyses, confirm that the following items are present in the figure legend, table legend, main text, or Methods section.

n/a	Confirmed	
[ ]	[x]	The exact sample size (*n*) for each experimental group/condition, given as a discrete number and unit of measurement
[ ]	[x]	A statement on whether measurements were taken from distinct samples or whether the same sample was measured repeatedly
[x]	[ ]	The statistical test(s) used AND whether they are one- or two-sided<br>*Only common tests should be described solely by name; describe more complex techniques in the Methods section.*
[x]	[ ]	A description of all covariates tested
[ ]	[x]	A description of any assumptions or corrections, such as tests of normality and adjustment for multiple comparisons
[ ]	[x]	A full description of the statistical parameters including central tendency (e.g. means) or other basic estimates (e.g. regression coefficient) AND variation (e.g. standard deviation) or associated estimates of uncertainty (e.g. confidence intervals)
[x]	[ ]	For null hypothesis testing, the test statistic (e.g. *F*, *t*, *r*) with confidence intervals, effect sizes, degrees of freedom and *P* value noted<br>*Give P values as exact values whenever suitable.*
[x]	[ ]	For Bayesian analysis, information on the choice of priors and Markov chain Monte Carlo settings
[x]	[ ]	For hierarchical and complex designs, identification of the appropriate level for tests and full reporting of outcomes
[x]	[ ]	Estimates of effect sizes (e.g. Cohen's *d*, Pearson's *r*), indicating how they were calculated

Our web collection on statistics for biologists contains articles on many of the points above.

Software and code

Policy information about availability of computer code

Data collection	No software was used for data collection.
Data analysis	Genetic map construction<br>Genetic maps were generated based on ordering markers relative to the consensus genetic map of barley. Validation of map order was based on recombination fraction plots in R/qtl (plot.rf; v1.42-8).<br><br>QTL analysis<br>QTL analysis was performed using QTL Cartographer (v1.17e) and the QKcartographer suite of Python scripts. All scripts are available on Github (https://github.com/matthewmoscou/QKcartographer). Marker-trait associations were performed using R/qtl (fitqtl; v1.42-8).<br><br>Transcriptome analysis<br>Raw RNAseq reads were trimmed using Trimmomatic (v0.32). De novo transcriptomes were assembled using Trinity (v2013-11-10). Alignment of reads to assembled transcripts was performed using bowtie2 (v2.3.4.1).<br><br>Sequence capture assembly<br>De novo assembly was performed using Geneious (v10.2.3) de novo assembly using custom sensitivity parameters for assembly: don't merge variants with coverage over approximately 6, merge homopolymer variants, allow gaps up to a maximum of 15% gaps per read, word length of 14, minimum overlap of 250 bp, ignore words repeated more than 200 times, 5% maximum mismatches per read, maximum gap size of 2, minimum overlap identity of 90%, index word length 12, reanalyze threshold of 8, and maximum ambiguity of 4.<br><br>Phylogenetic analysis<br>Alignment of RNAseq reads to the reference barley gene set was performed using bwa mem (version 0.7.5a-r405). SAM and BAM files were

generated using samtools (version 0.1.19-96b5f2294a). Coverage was determined using bedtools (version v2.17.0). SNPs and InDels were called using VarScan (version 2.3.8). The QKgenome suite (version 1.1.1) was used to assess allelic diversity in barley coding sequence among diverse genotypes. QKgenome_conversion.py was used to evaluate nucleotide variation. A multiple sequence alignment of polymorphic sites was generated using QKgenome_phylogeny.py (v1.0). The phylogenetic tree was constructed with RAxML (v8.2.9). All scripts are available on Github (https://github.com/matthewmoscou/QKgenome and https://github.com/matthewmoscou/QKphylogeny).

For manuscripts utilizing custom algorithms or software that are central to the research but not yet described in published literature, software must be made available to editors and reviewers. We strongly encourage code deposition in a community repository (e.g. GitHub). See the Nature Portfolio guidelines for submitting code & software for further information.

Data

Policy information about availability of data

All manuscripts must include a data availability statement. This statement should provide the following information, where applicable:

- Accession codes, unique identifiers, or web links for publicly available datasets
- A description of any restrictions on data availability
- For clinical datasets or third party data, please ensure that the statement adheres to our policy

The RNAseq data generated in this study have been deposited in the NCBI database under BioProject codes PRJNA292371, PRJNA376252, PRJNA378334, and PRJNA378723. The NLR gene captures of barley accessions CI 16153 and Golden Promise generated in this study have been deposited in the NCBI database under BioProject codes PRJNA523805 and PRJNA523807. The sequences of plasmids used for plant transformation in this study have been deposited in the NCBI database with accession codes MZ555767 (p6:Mla8:t6), MZ555768 (p6:Mla1:t6), and MZ555769 (p6:Mla6:t6) and Figshare. Genotypic, phenotypic, and source data for all figures and supplementary figures have been deposited on Figshare. The QKcartographer, QKgenome, and QKphylogeny suite of Python scripts are maintained on GitHub (https://github.com/matthewmoscou/QKcartographer; https://github.com/matthewmoscou/QKgenome; https://github.com/matthewmoscou/QKphylogeny) and Figshare.

Field-specific reporting

Please select the one below that is the best fit for your research. If you are not sure, read the appropriate sections before making your selection.

[x] Life sciences [ ] Behavioural & social sciences [ ] Ecological, evolutionary & environmental sciences

For a reference copy of the document with all sections, see nature.com/documents/nr-reporting-summary-flat.pdf

Life sciences study design

All studies must disclose on these points even when the disclosure is negative.

Sample size	All mapping population sizes are described in Supplemental Table 5. Eight seedlings were screened for individual parental accessions, doubled-haploid populations, and recombinant inbred line populations. A general requirement of ~90 or more individuals was required for F2 and BC1 populations. For F2 populations, a sample size of ~90 individuals was determined as a minimum number for statistical significane for two segregating dominant resistance genes (as individual genes segregate 3:1 for dominant resistance genes). A balance between breadth versus depth sampling of diversity. BC1 populations were used, when possible, due to the complex genetic architecture underlying wheat stripe rust resistance in barley, as individual genes would segregate at a 1:1 ratio (dominant resistance genes). Pathogen assays with stripe rust are robust over temporal data sets in controlled environment rooms.
Data exclusions	No data was excluded.
Replication	All parental accessions were evaluated in two biological replicates with the evaluation of an average of eight seedlings. Two biological replicates were performed for all doubled-haploid and recombinant inbred line populations using an average of eight seedlings, whereas single experiments were performed with F2 and BC1 populations. Transgenic T1 and T2 families were evaluated at least once with individual stripe rust isolates (twice for Pst isolate 08/21). Digital droplet PCR was performed on three to four biological replicates, except for no replication on transgenic lines. All attempts at replication were successful for all experiments described. For experiments involving Manchuria near-isogenic lines and a subset of the SusPtrit x Golden Promise doubled haploid population inoculated with wheat stripe rust isolate 16/035, three independent biological replicates were assessed.
Randomization	All plants were allocated in random complete design in individual experiments.
Blinding	During data collection, individuals scoring phenotypes of plants were unaware of the genotype. During data analysis, no blinding was used, as prior knowledge of the genotype-phenotype relationship was necessary for data analysis.

Reporting for specific materials, systems and methods

We require information from authors about some types of materials, experimental systems and methods used in many studies. Here, indicate whether each material, system or method listed is relevant to your study. If you are not sure if a list item applies to your research, read the appropriate section before selecting a response.

Materials & experimental systems

n/a	Involved in the study
☒	☐ Antibodies
☒	☐ Eukaryotic cell lines
☒	☐ Palaeontology and archaeology
☒	☐ Animals and other organisms
☒	☐ Human research participants
☒	☐ Clinical data
☒	☐ Dual use research of concern

Methods

n/a	Involved in the study
☒	☐ ChIP-seq
☒	☐ Flow cytometry
☒	☐ MRI-based neuroimaging
