# Supplementary figures and images for: The barley immune receptor Mla recognizes multiple pathogens and contributes to host range dynamics
Source: Nat Commun. 2021 Nov 25;12:6915. doi: 10.1038/s41467-021-27288-3 (PMC8617247; doi:10.1038/s41467-021-27288-3)

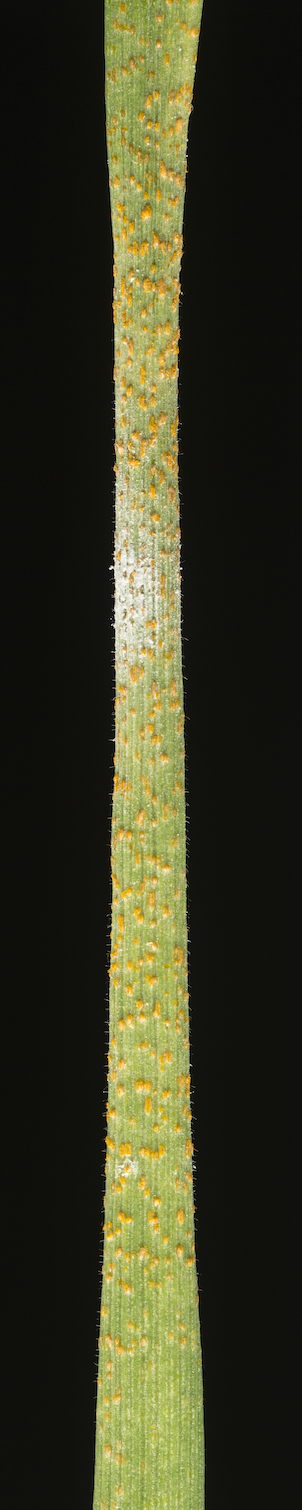

Supplement: Supplementary file 16 — Source Data [file 41467_2021_27288_MOESM16_ESM.zip › Source Data/Figure 6/cropped_images/DSC_7601_Chinese_Spring_cropped_2.TIF]

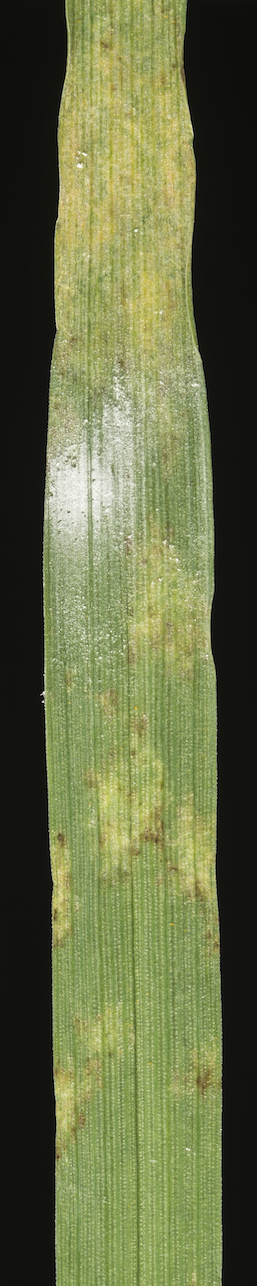

Supplement: Supplementary file 16 — Source Data [file 41467_2021_27288_MOESM16_ESM.zip › Source Data/Figure 6/cropped_images/DSC_7612_HVT_00074_hemizygous_cropped_2.tif]

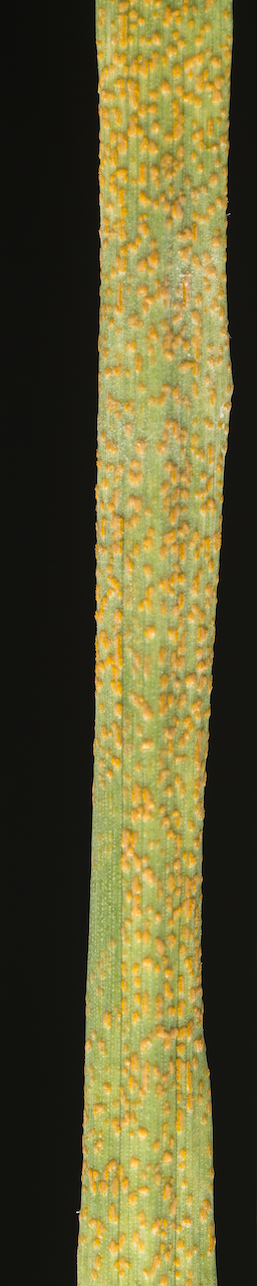

Supplement: Supplementary file 16 — Source Data [file 41467_2021_27288_MOESM16_ESM.zip › Source Data/Figure 6/cropped_images/DSC_7612_HVT_00074_hemizygous_cropped_3.tif]

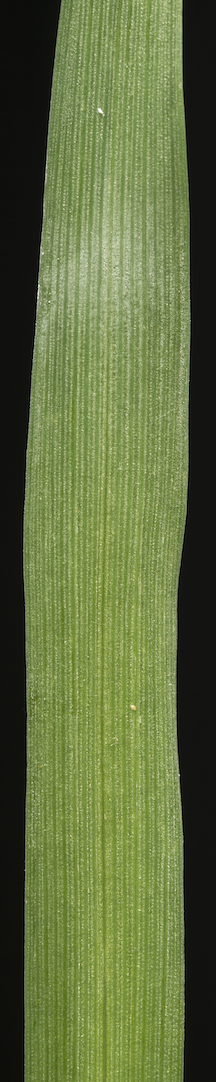

Supplement: Supplementary file 16 — Source Data [file 41467_2021_27288_MOESM16_ESM.zip › Source Data/Figure 6/cropped_images/DSC_7605_GoldenPromise_cropped_3.tif]

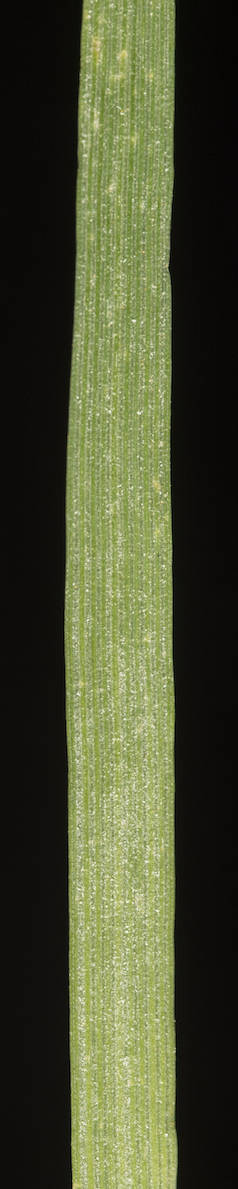

Supplement: Supplementary file 16 — Source Data [file 41467_2021_27288_MOESM16_ESM.zip › Source Data/Figure 6/cropped_images/_DSC4288_ChineseSpring_cropped_4.tif]

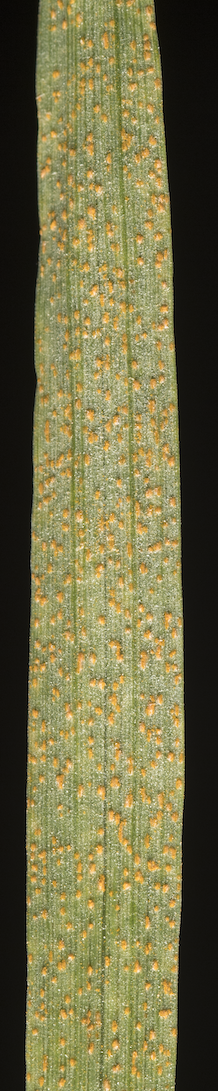

Supplement: Supplementary file 16 — Source Data [file 41467_2021_27288_MOESM16_ESM.zip › Source Data/Figure 6/cropped_images/_DSC4303_HVT_00072_null_cropped_2.tif]

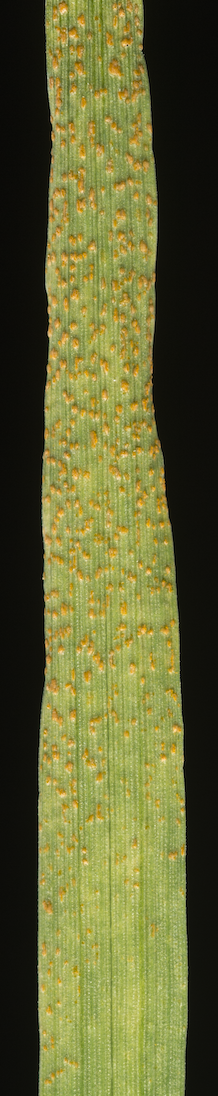

Supplement: Supplementary file 16 — Source Data [file 41467_2021_27288_MOESM16_ESM.zip › Source Data/Figure 6/cropped_images/DSC_7603_SxGP_DH-47_cropped_1.tif]

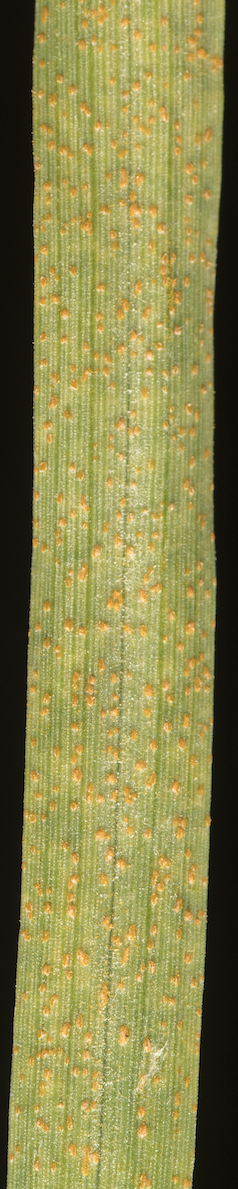

Supplement: Supplementary file 16 — Source Data [file 41467_2021_27288_MOESM16_ESM.zip › Source Data/Figure 6/cropped_images/_DSC4292_SxGP_DH-47_cropped_4.tif]

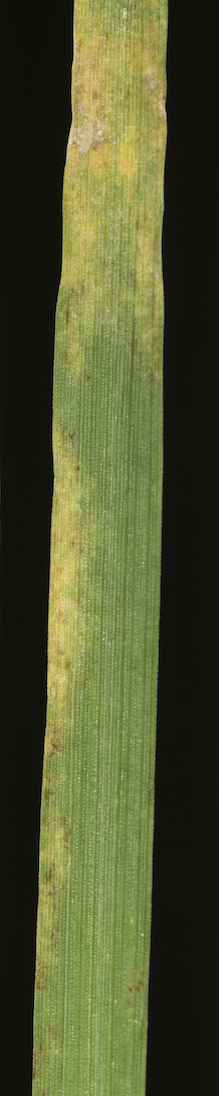

Supplement: Supplementary file 16 — Source Data [file 41467_2021_27288_MOESM16_ESM.zip › Source Data/Figure 6/cropped_images/DSC_7613_HVT_00111_hemizygous_cropped_4.tif]

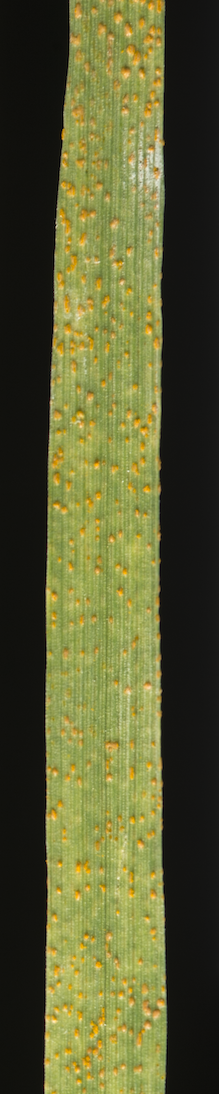

Supplement: Supplementary file 16 — Source Data [file 41467_2021_27288_MOESM16_ESM.zip › Source Data/Figure 6/cropped_images/DSC_7613_HVT_00111_hemizygous_cropped_5.tif]

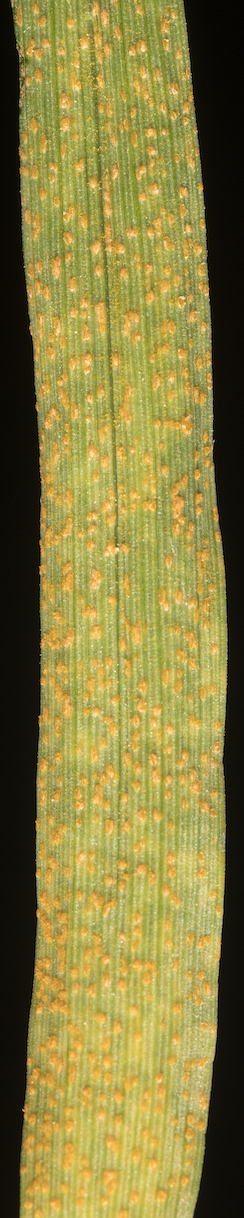

Supplement: Supplementary file 16 — Source Data [file 41467_2021_27288_MOESM16_ESM.zip › Source Data/Figure 6/cropped_images/_DSC4295_GoldenPromise_cropped_3.tif]

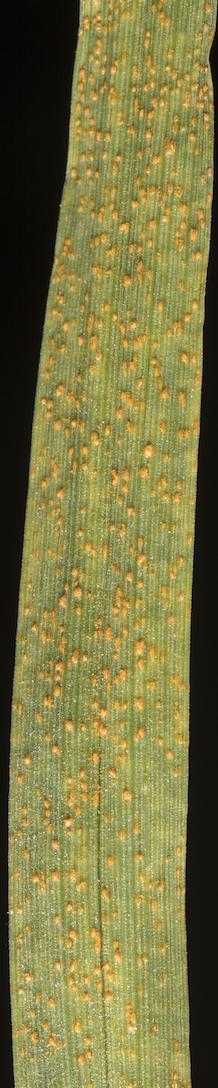

Supplement: Supplementary file 16 — Source Data [file 41467_2021_27288_MOESM16_ESM.zip › Source Data/Figure 6/cropped_images/_DSC4306_HVT_00111_hemizygous_cropped_2.tif]

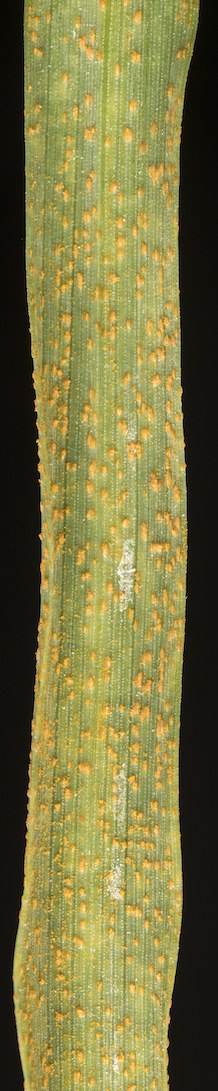

Supplement: Supplementary file 16 — Source Data [file 41467_2021_27288_MOESM16_ESM.zip › Source Data/Figure 6/cropped_images/_DSC4305_HVT_00074_hemizygous_cropped_2.tif]

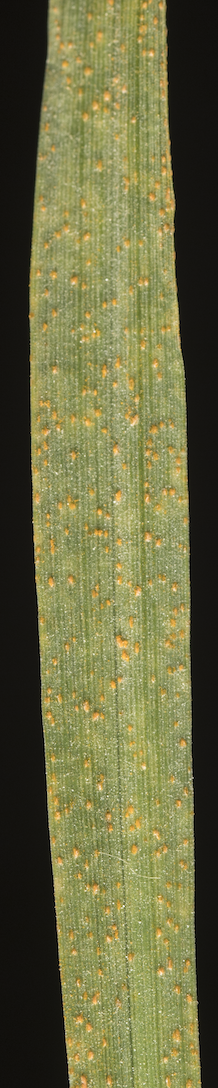

Supplement: Supplementary file 16 — Source Data [file 41467_2021_27288_MOESM16_ESM.zip › Source Data/Figure 6/cropped_images/_DSC4306_HVT_00111_hemizygous_cropped_3.tif]

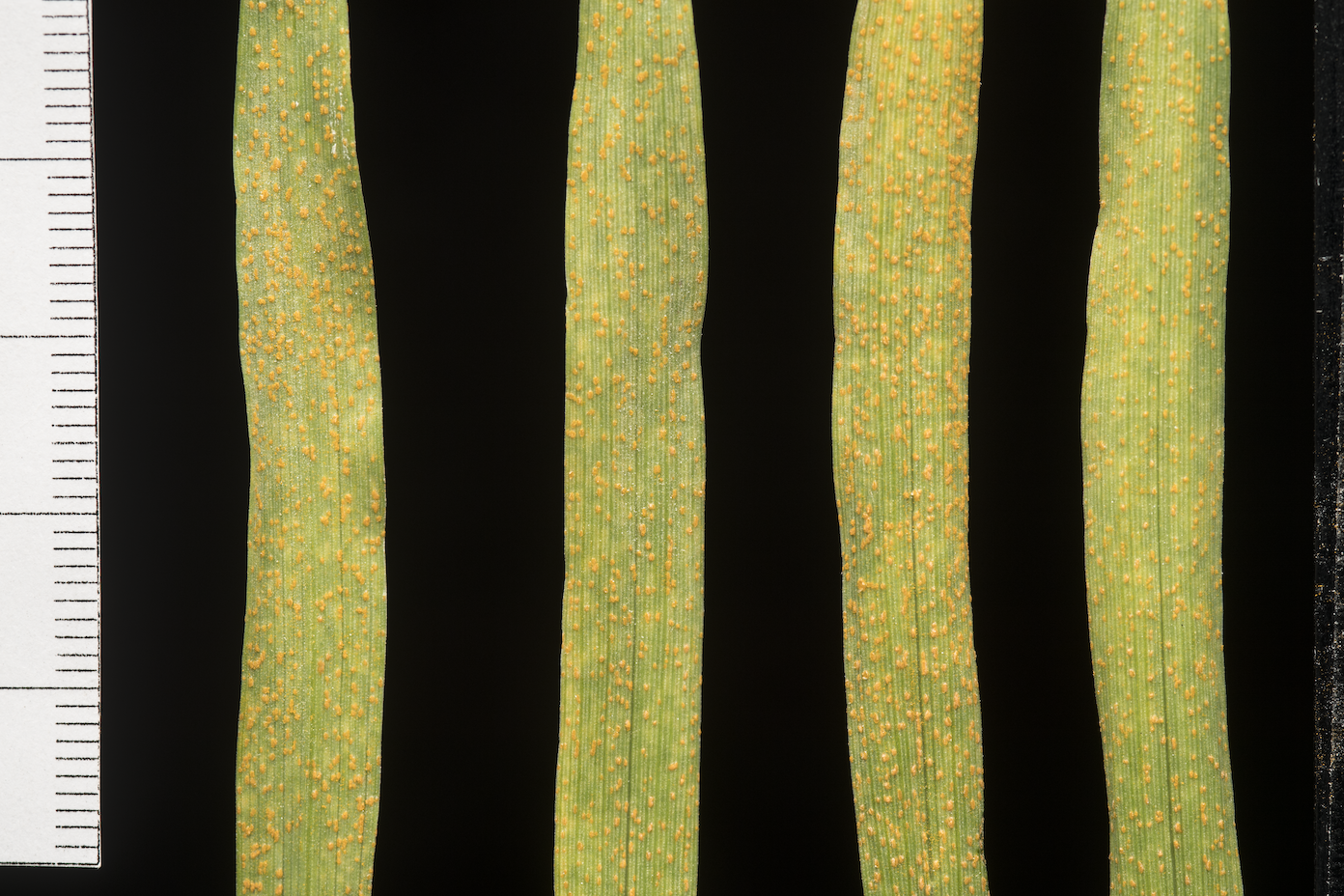

Supplement: Supplementary file 16 — Source Data [file 41467_2021_27288_MOESM16_ESM.zip › Source Data/Figure 6/raw_images/_DSC4295_GoldenPromise.TIF]

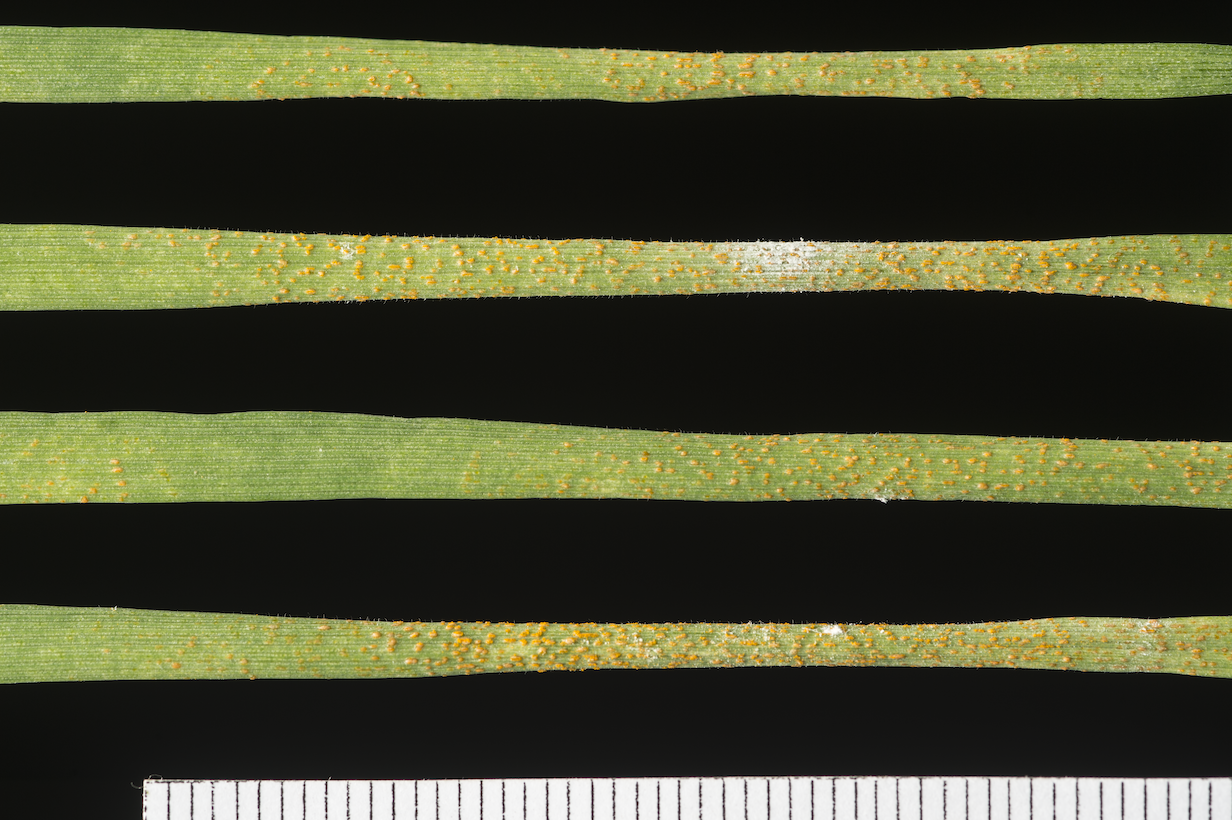

Supplement: Supplementary file 16 — Source Data [file 41467_2021_27288_MOESM16_ESM.zip › Source Data/Figure 6/raw_images/DSC_7601_Chinese_Spring.TIF]

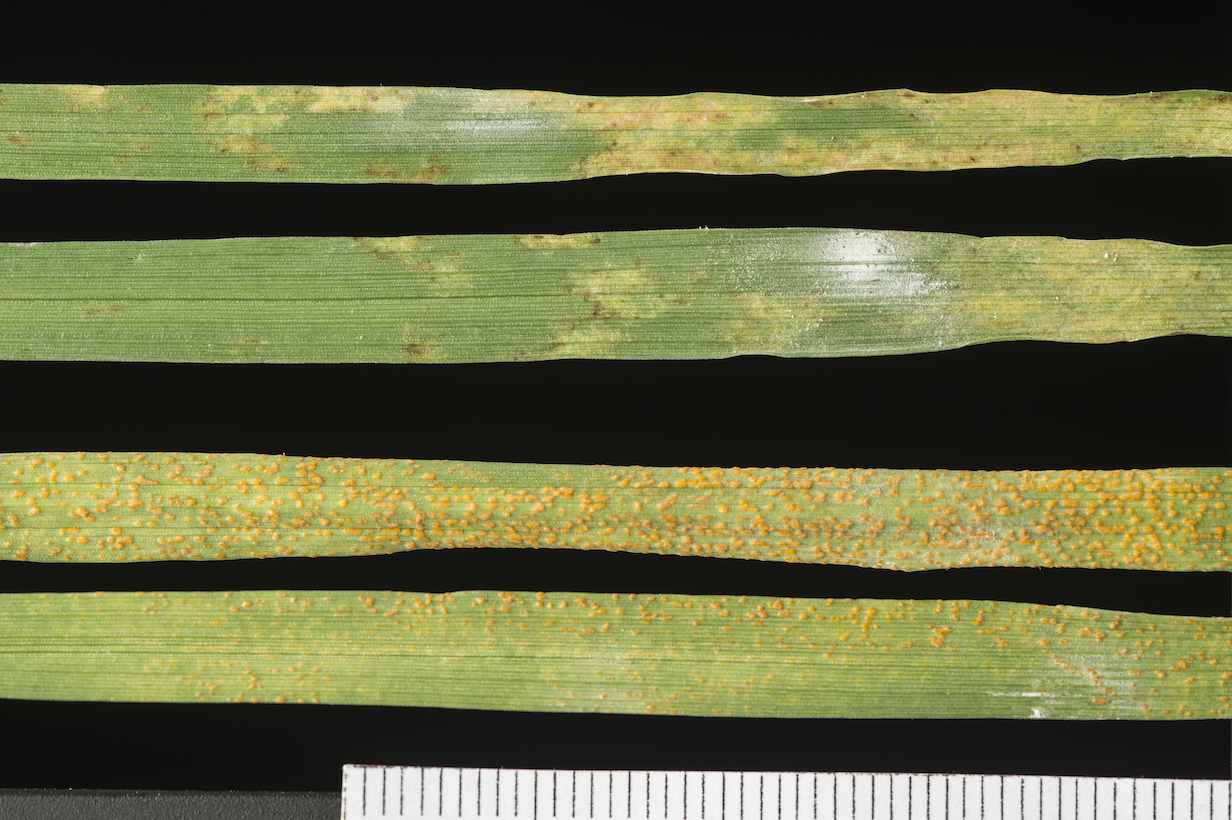

Supplement: Supplementary file 16 — Source Data [file 41467_2021_27288_MOESM16_ESM.zip › Source Data/Figure 6/raw_images/DSC_7612_HVT_00074_hemizygous.TIF]

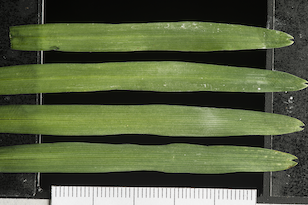

Supplement: Supplementary file 16 — Source Data [file 41467_2021_27288_MOESM16_ESM.zip › Source Data/Figure 6/raw_images/DSC_7605_GoldenPromise.TIF]

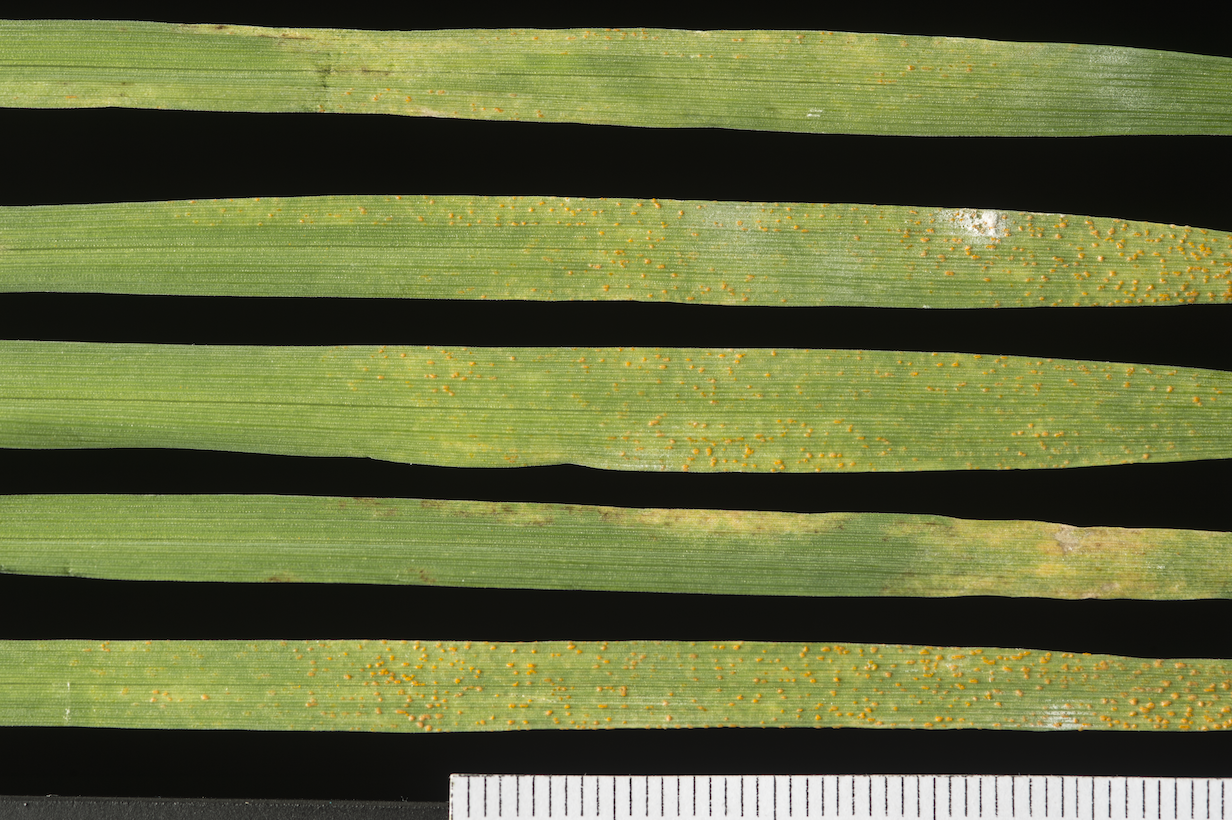

Supplement: Supplementary file 16 — Source Data [file 41467_2021_27288_MOESM16_ESM.zip › Source Data/Figure 6/raw_images/DSC_7613_HVT_00111_hemizygous.TIF]

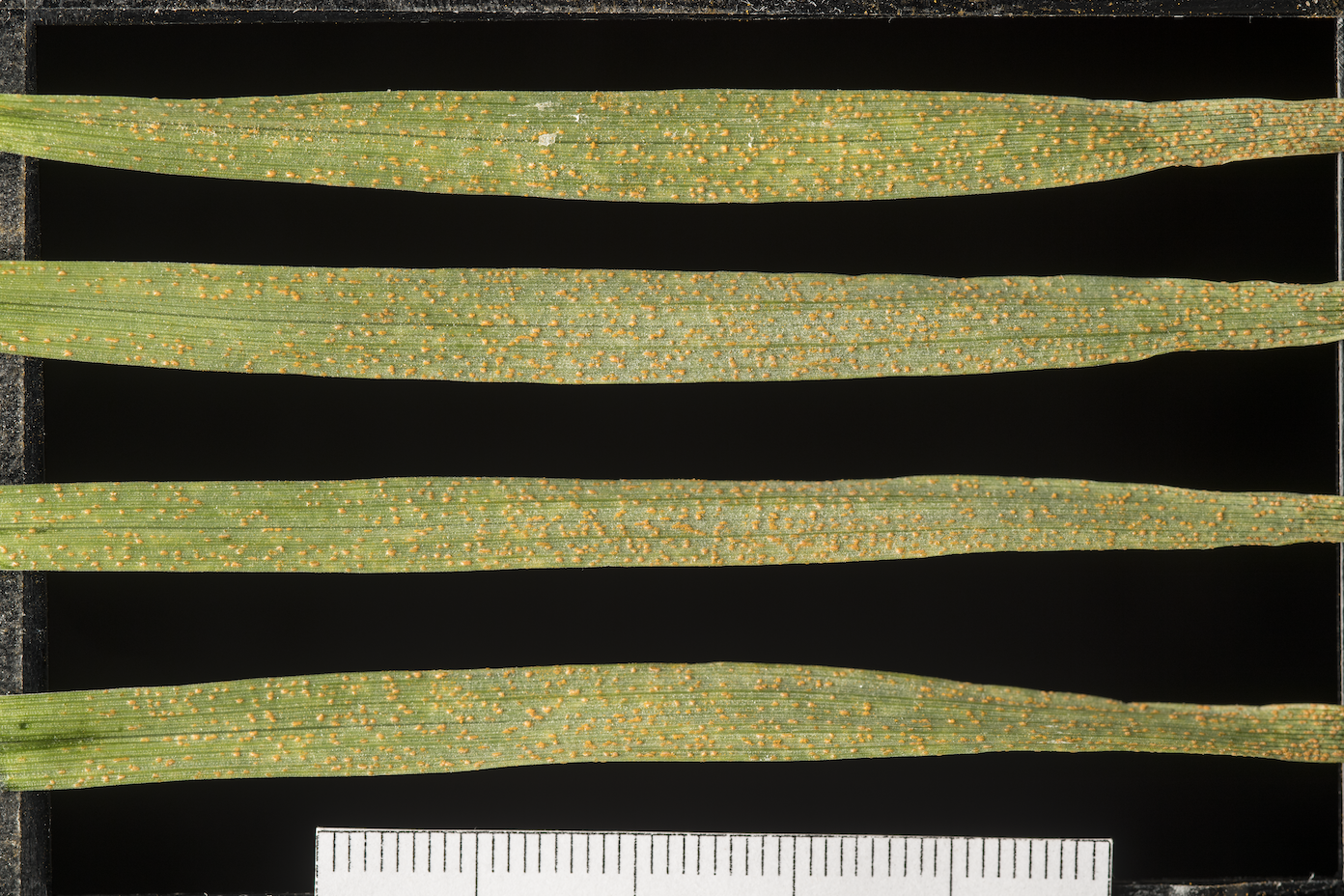

Supplement: Supplementary file 16 — Source Data [file 41467_2021_27288_MOESM16_ESM.zip › Source Data/Figure 6/raw_images/_DSC4303_HVT_00072_null.TIF]

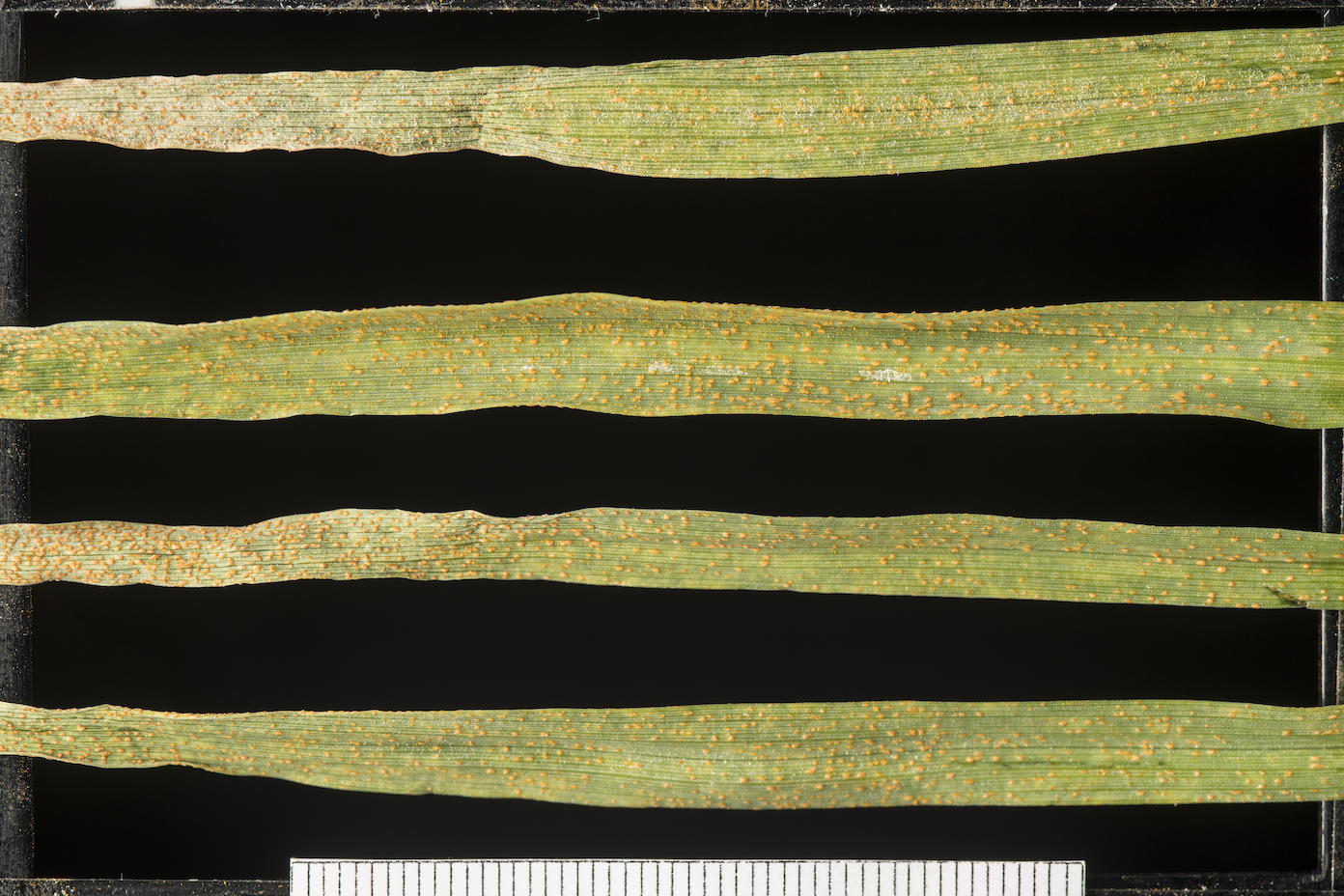

Supplement: Supplementary file 16 — Source Data [file 41467_2021_27288_MOESM16_ESM.zip › Source Data/Figure 6/raw_images/_DSC4305_HVT_00074_hemizygous.TIF]

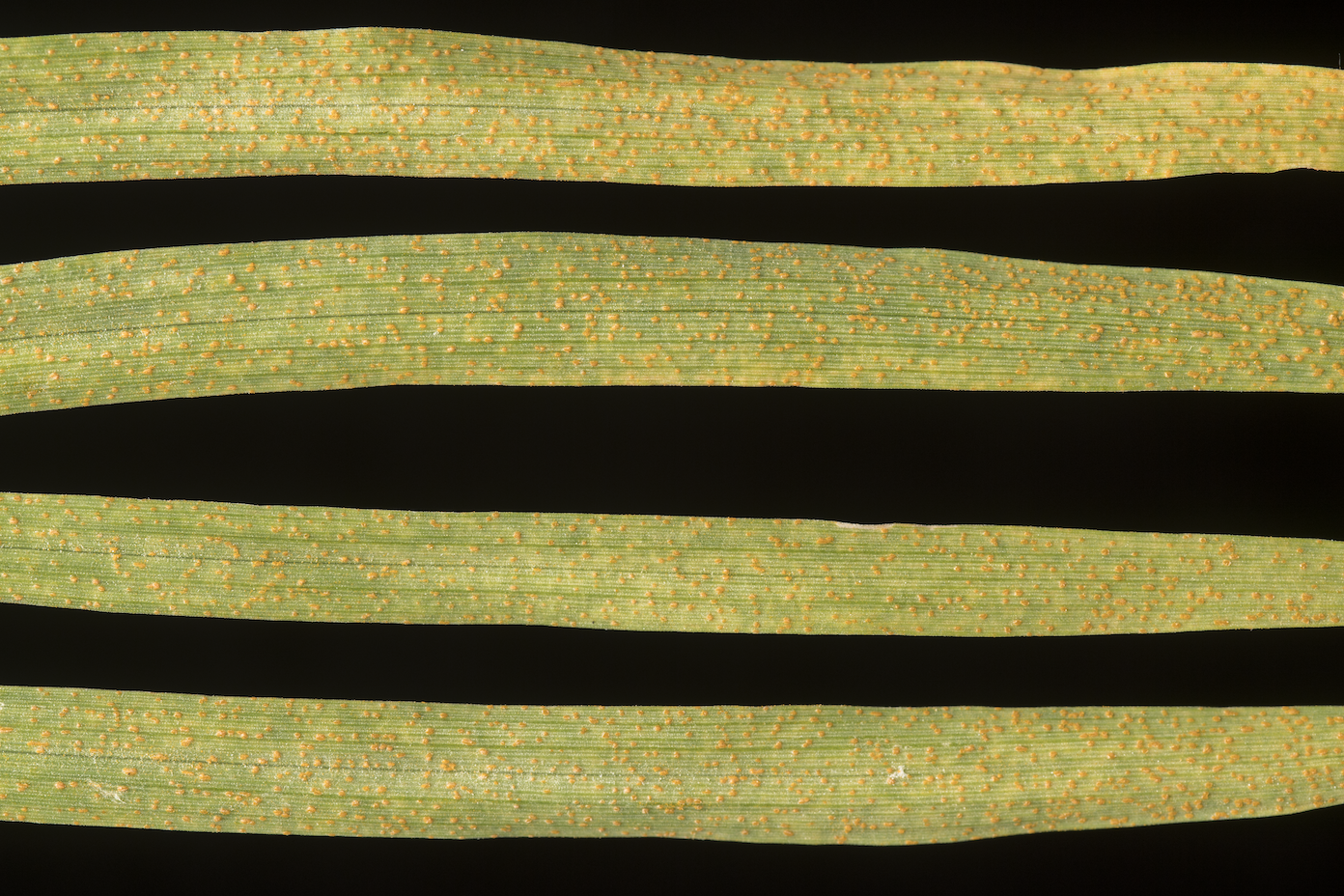

Supplement: Supplementary file 16 — Source Data [file 41467_2021_27288_MOESM16_ESM.zip › Source Data/Figure 6/raw_images/_DSC4292_SxGP_DH-47.TIF]

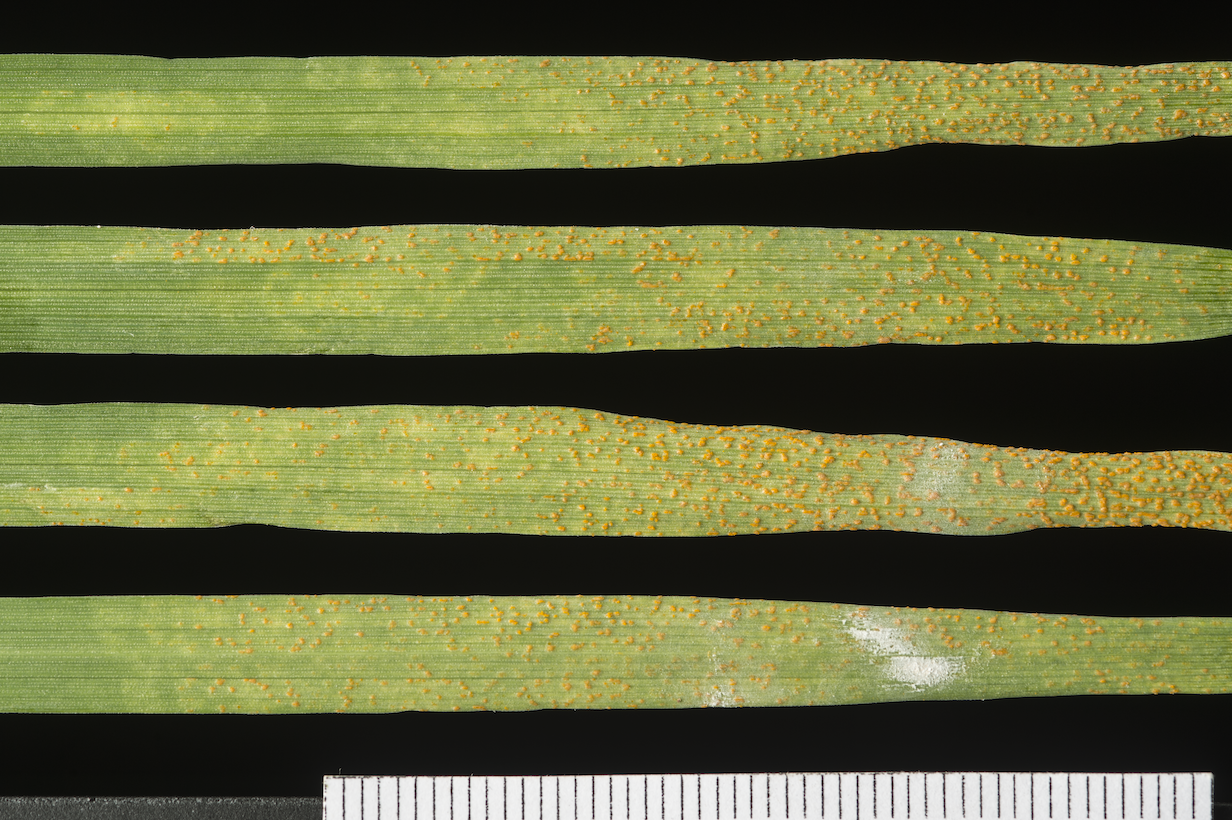

Supplement: Supplementary file 16 — Source Data [file 41467_2021_27288_MOESM16_ESM.zip › Source Data/Figure 6/raw_images/DSC_7603_SxGP_DH-47.TIF]

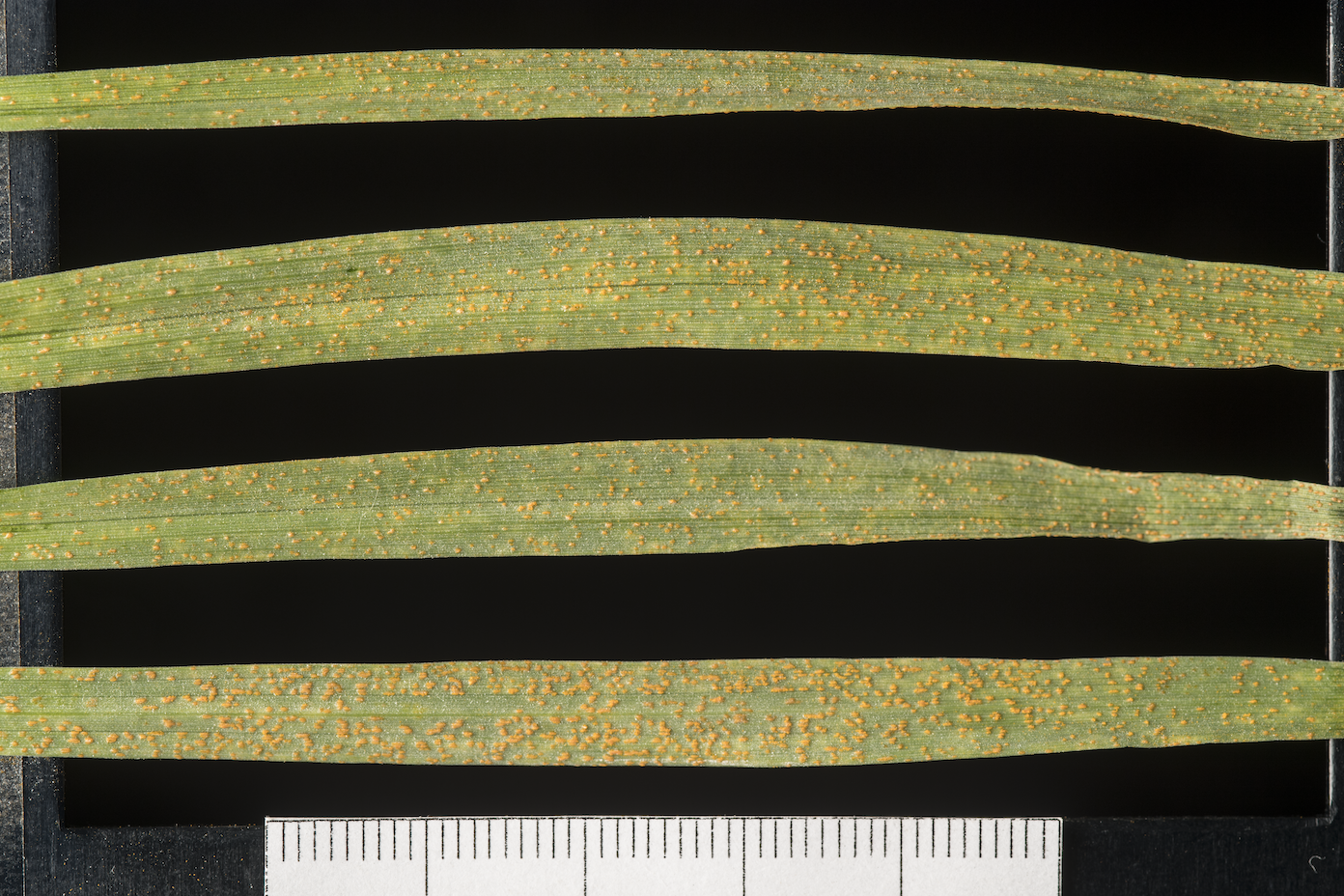

Supplement: Supplementary file 16 — Source Data [file 41467_2021_27288_MOESM16_ESM.zip › Source Data/Figure 6/raw_images/_DSC4306_HVT_00111_hemizygous.TIF]

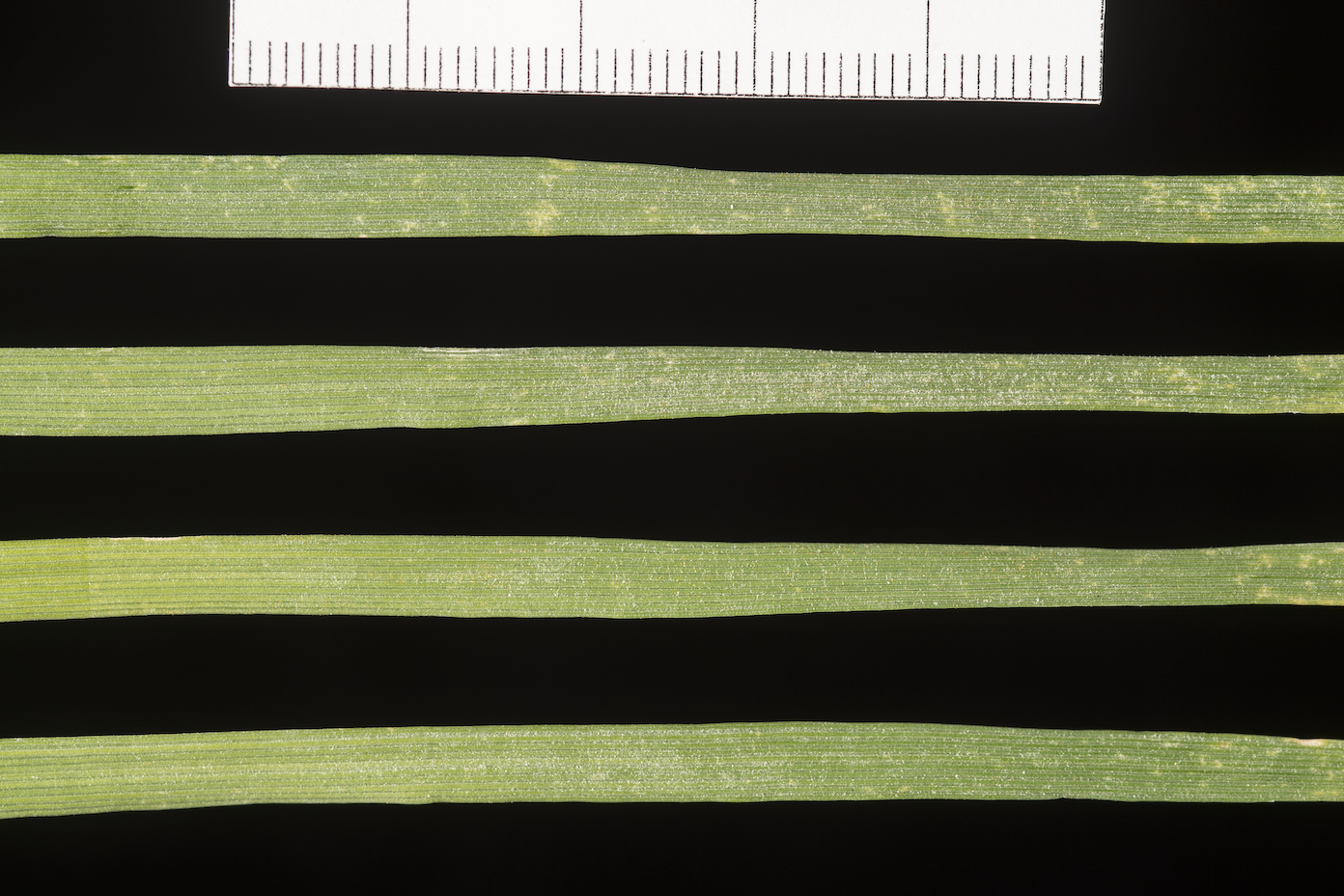

Supplement: Supplementary file 16 — Source Data [file 41467_2021_27288_MOESM16_ESM.zip › Source Data/Figure 6/raw_images/_DSC4288_ChineseSpring.TIF]

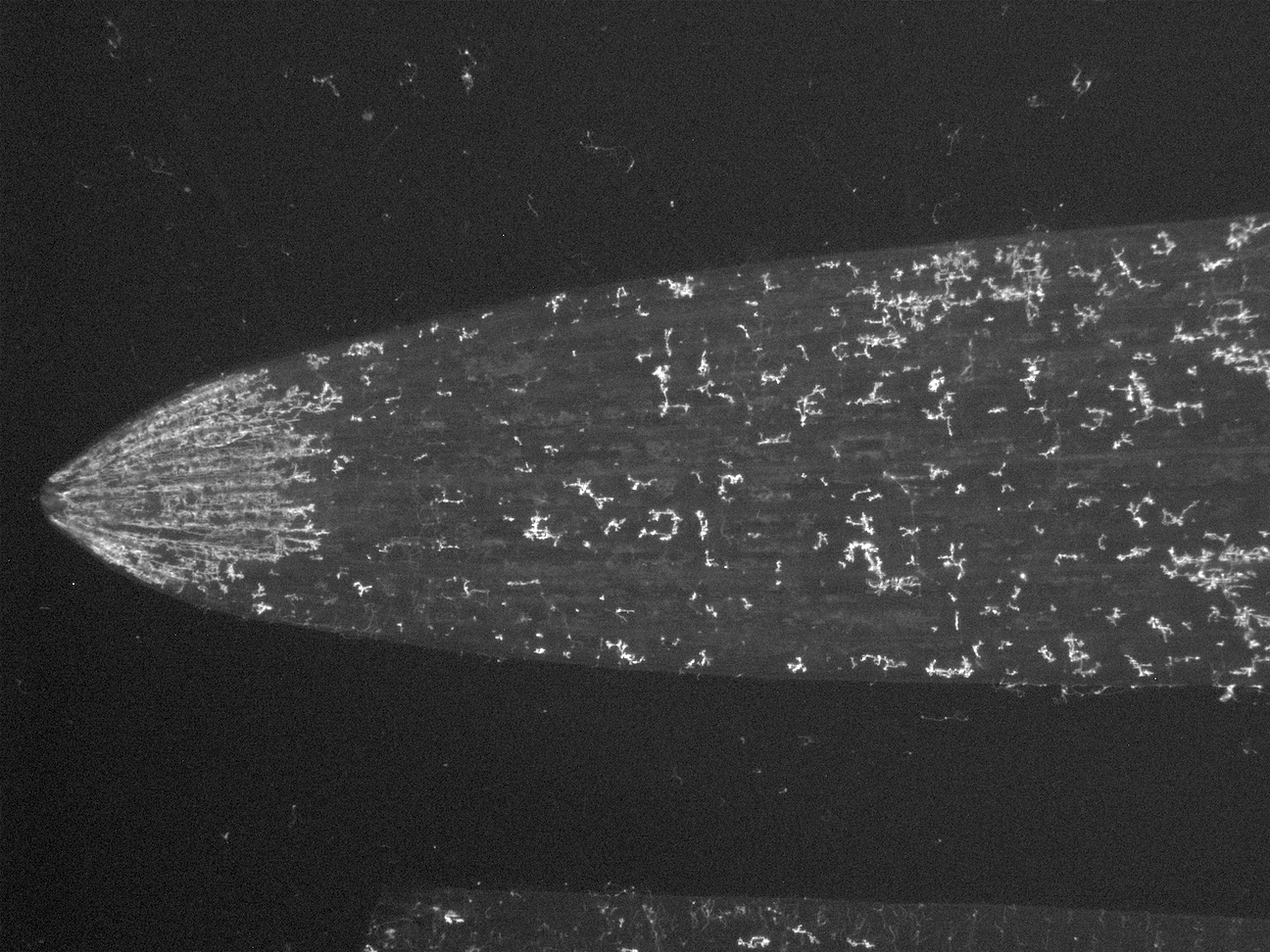

Supplement: Supplementary file 16 — Source Data [file 41467_2021_27288_MOESM16_ESM.zip › Source Data/Figure 1/micrographs/MM_687-300_L_SxGP_DH-65.tif]

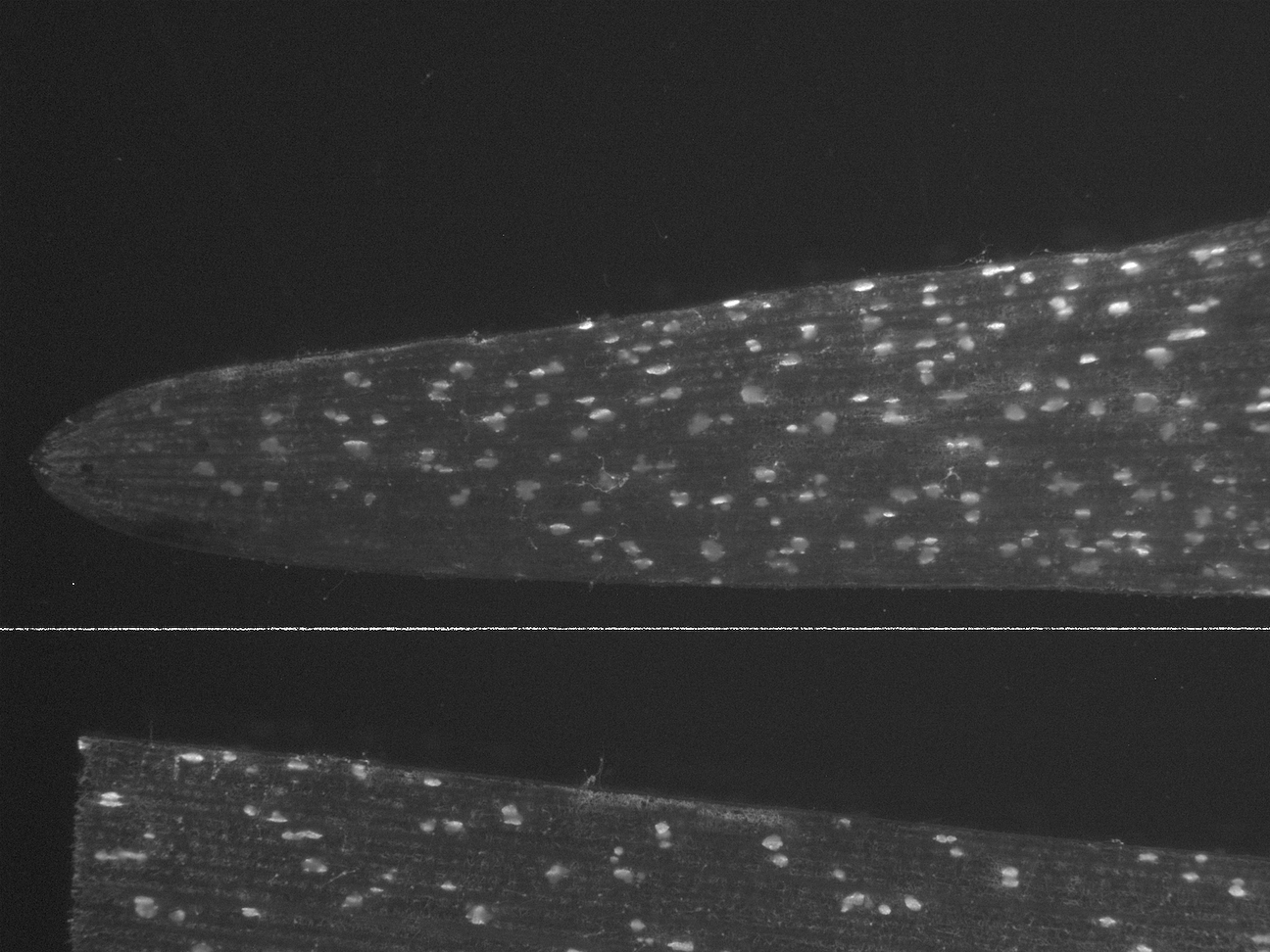

Supplement: Supplementary file 16 — Source Data [file 41467_2021_27288_MOESM16_ESM.zip › Source Data/Figure 1/micrographs/MM_687-234A_SusPtrit.tif]

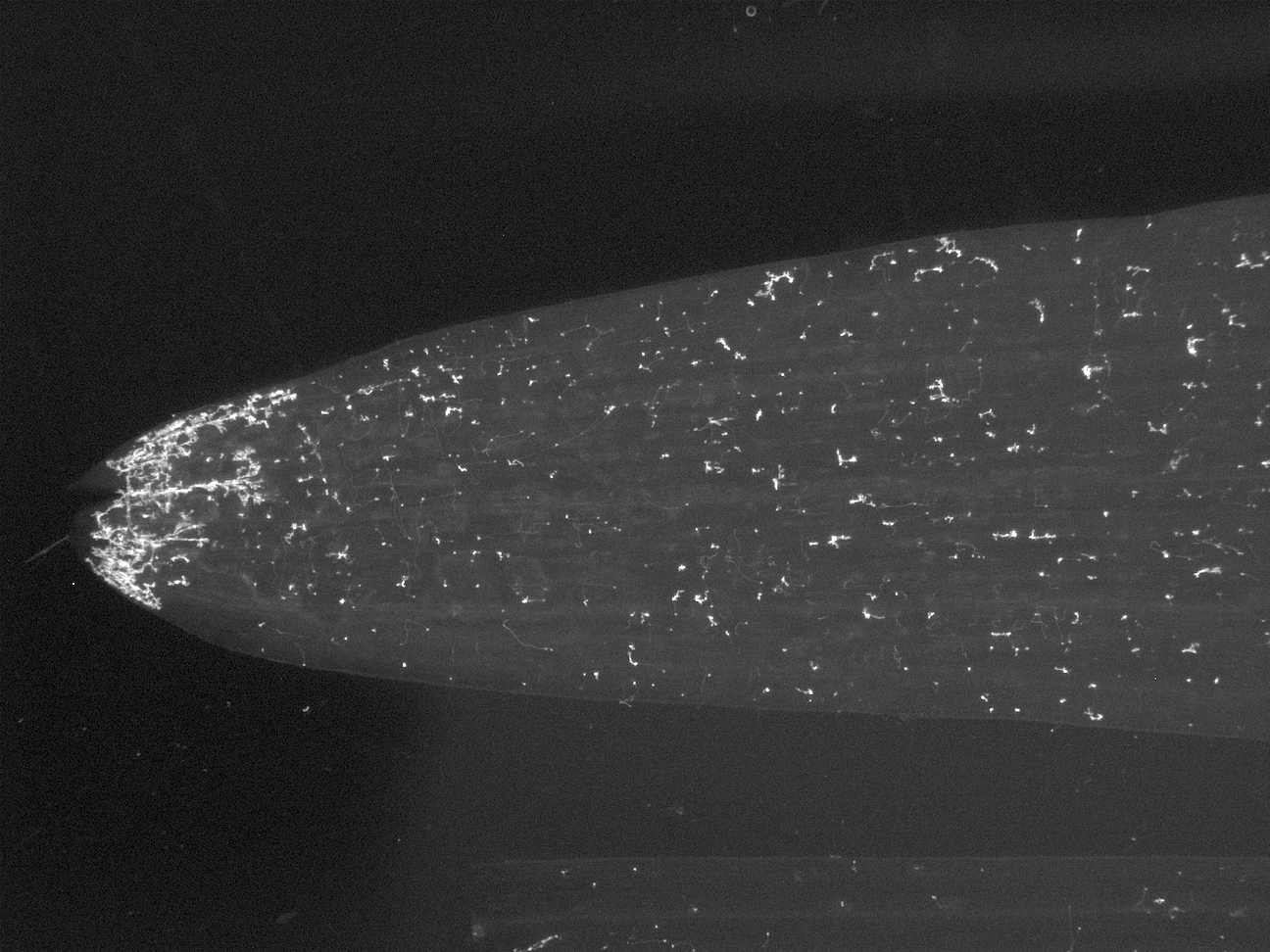

Supplement: Supplementary file 16 — Source Data [file 41467_2021_27288_MOESM16_ESM.zip › Source Data/Figure 1/micrographs/MM_687-235B_L_GoldenPromise.tif]

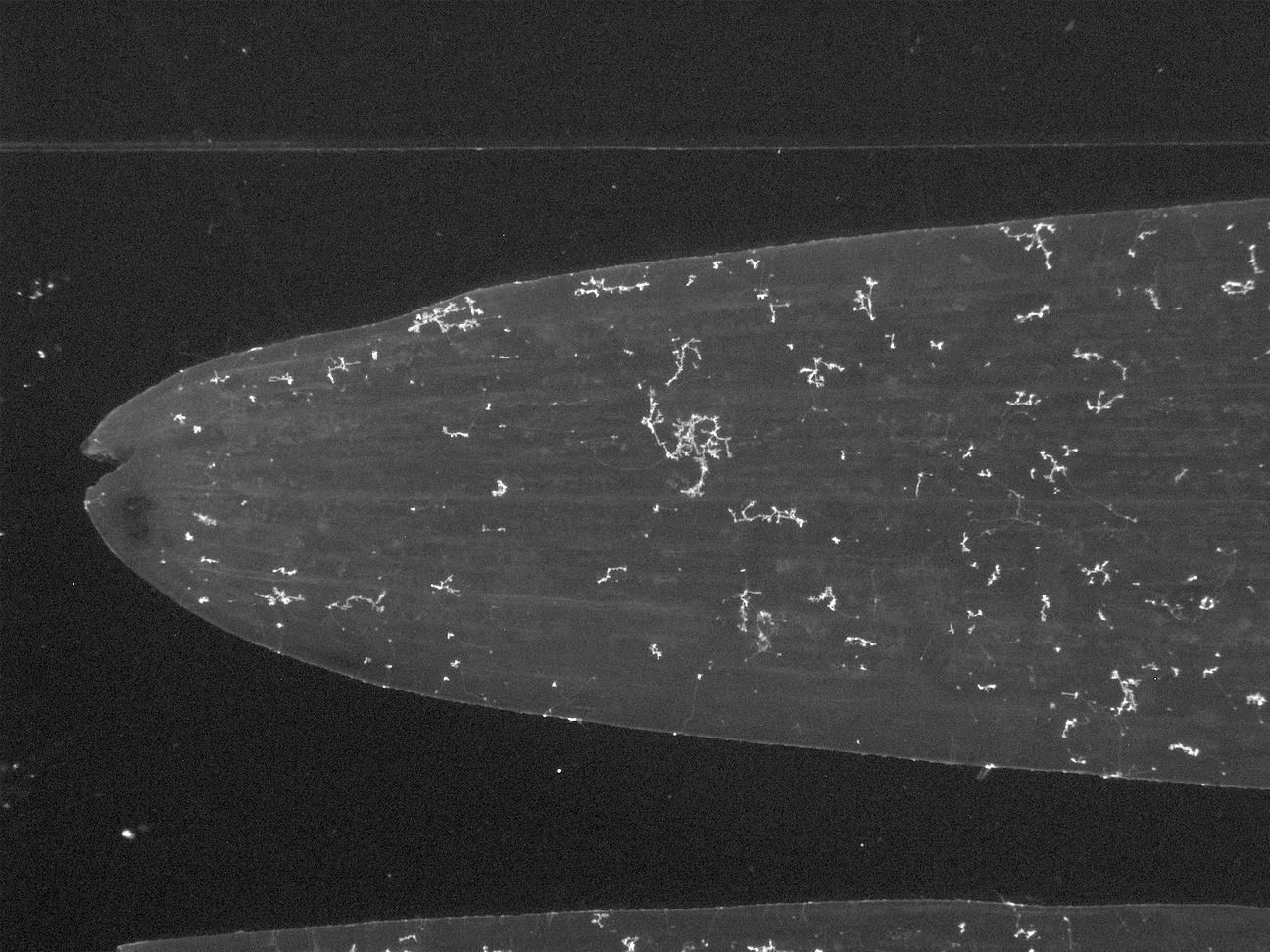

Supplement: Supplementary file 16 — Source Data [file 41467_2021_27288_MOESM16_ESM.zip › Source Data/Figure 1/micrographs/MM_687-302B_L_SxGP_DH-67.tif]

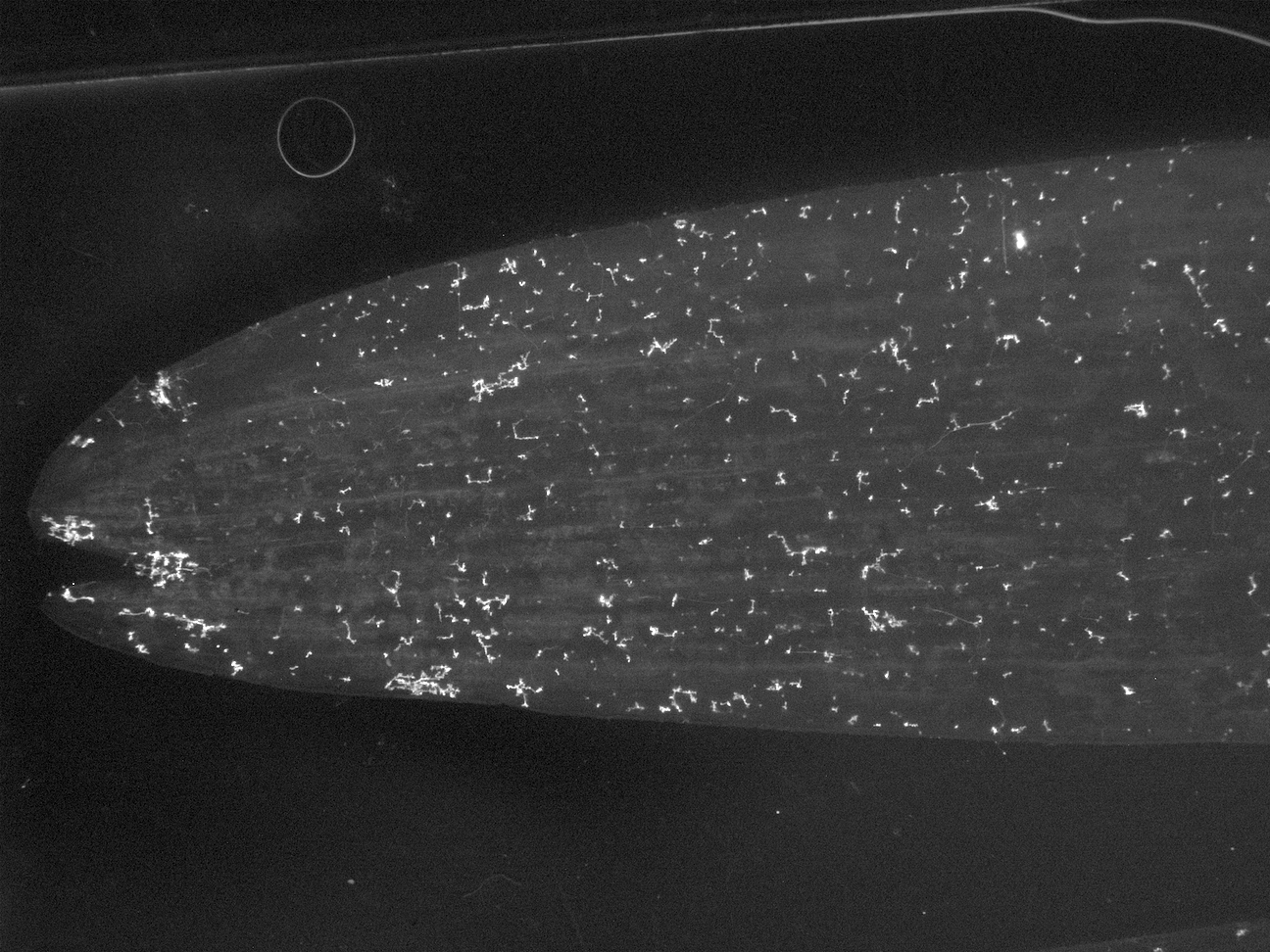

Supplement: Supplementary file 16 — Source Data [file 41467_2021_27288_MOESM16_ESM.zip › Source Data/Figure 1/micrographs/MM_0687-269A_L_SxGP_DH-34.tif]

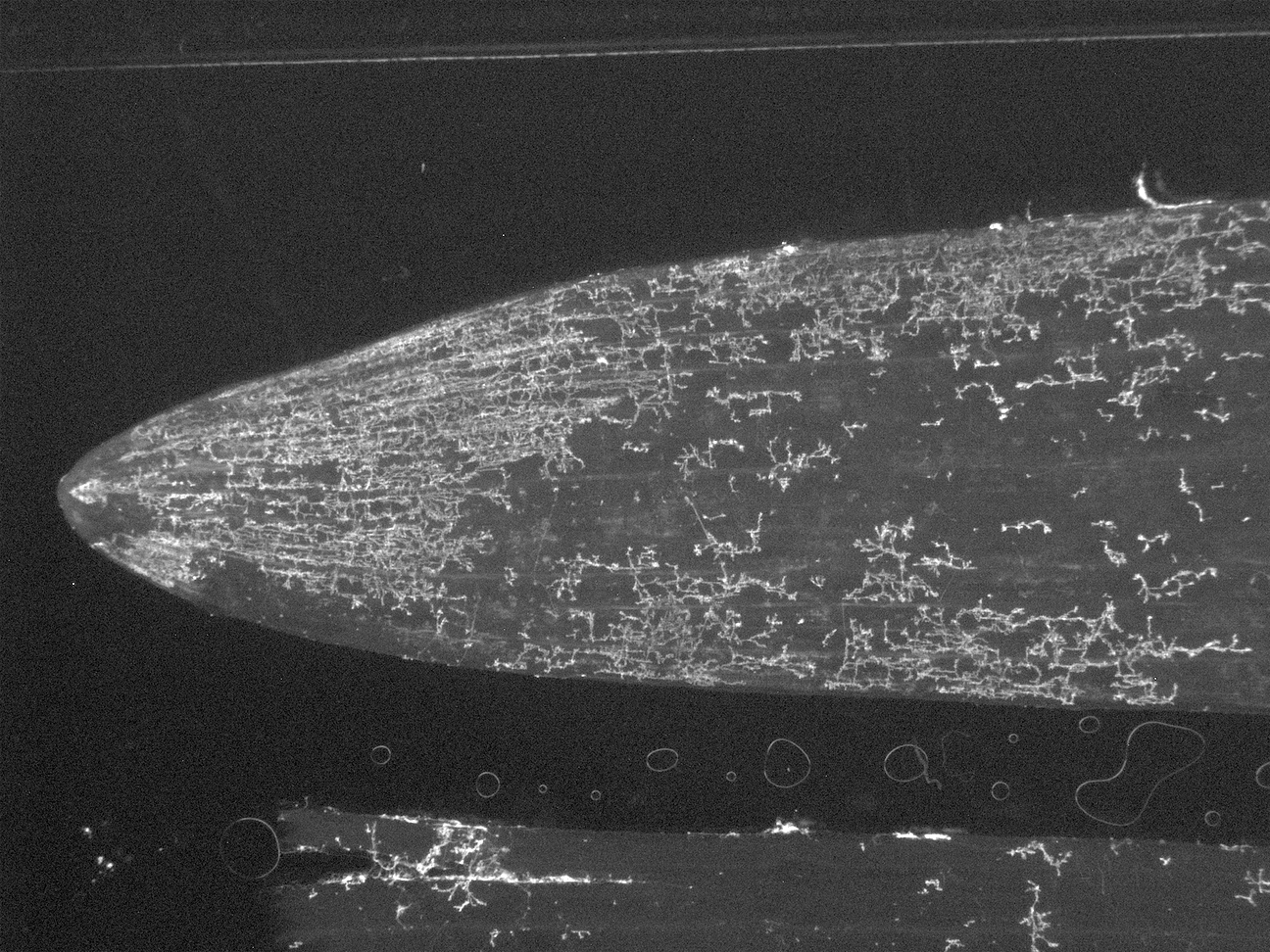

Supplement: Supplementary file 16 — Source Data [file 41467_2021_27288_MOESM16_ESM.zip › Source Data/Figure 1/micrographs/MM_687-252A_L_SxGP_DH-17.tif]

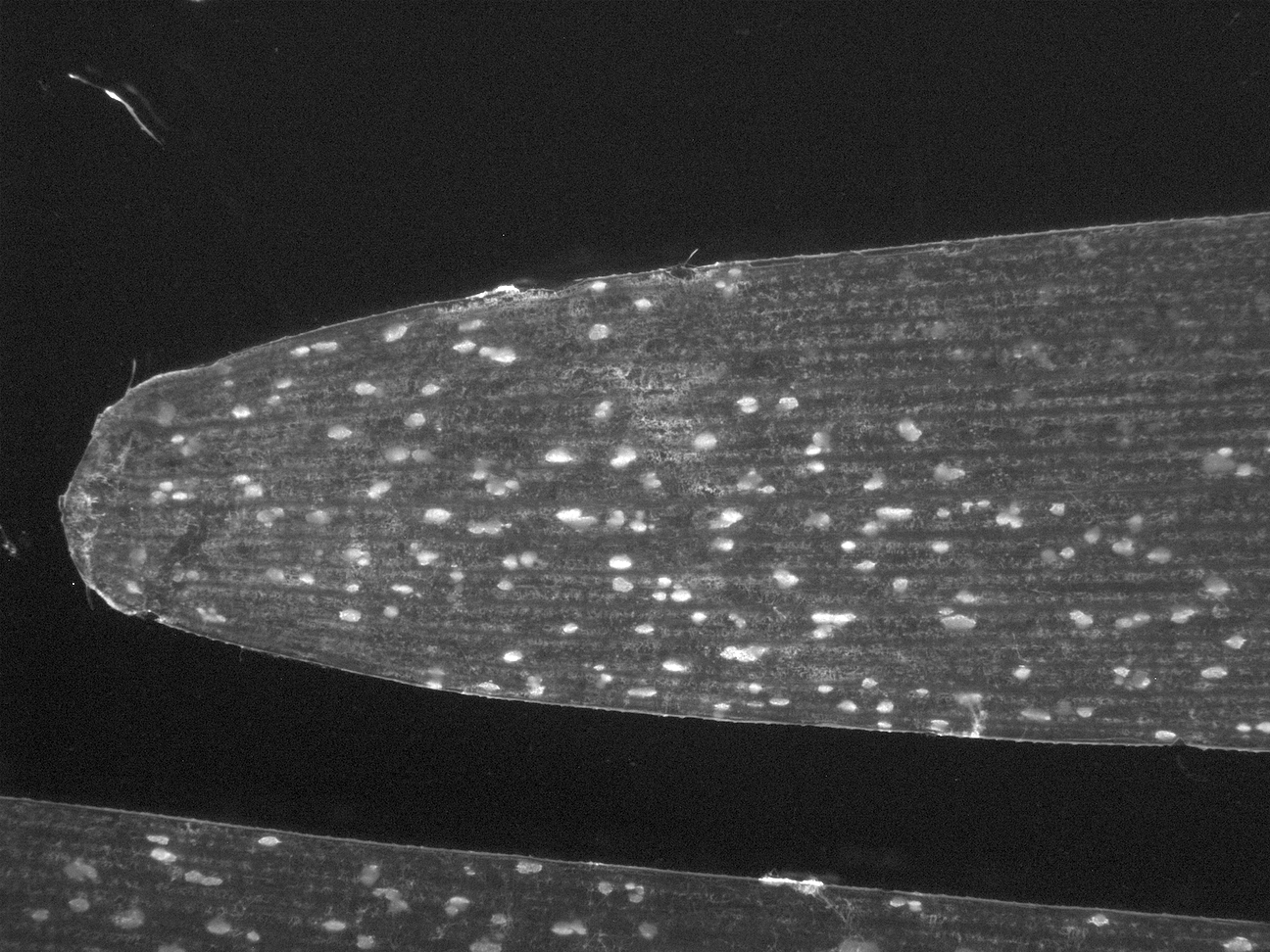

Supplement: Supplementary file 16 — Source Data [file 41467_2021_27288_MOESM16_ESM.zip › Source Data/Figure 1/micrographs/MM_687-303B_L_SxGP_DH-68.tif]

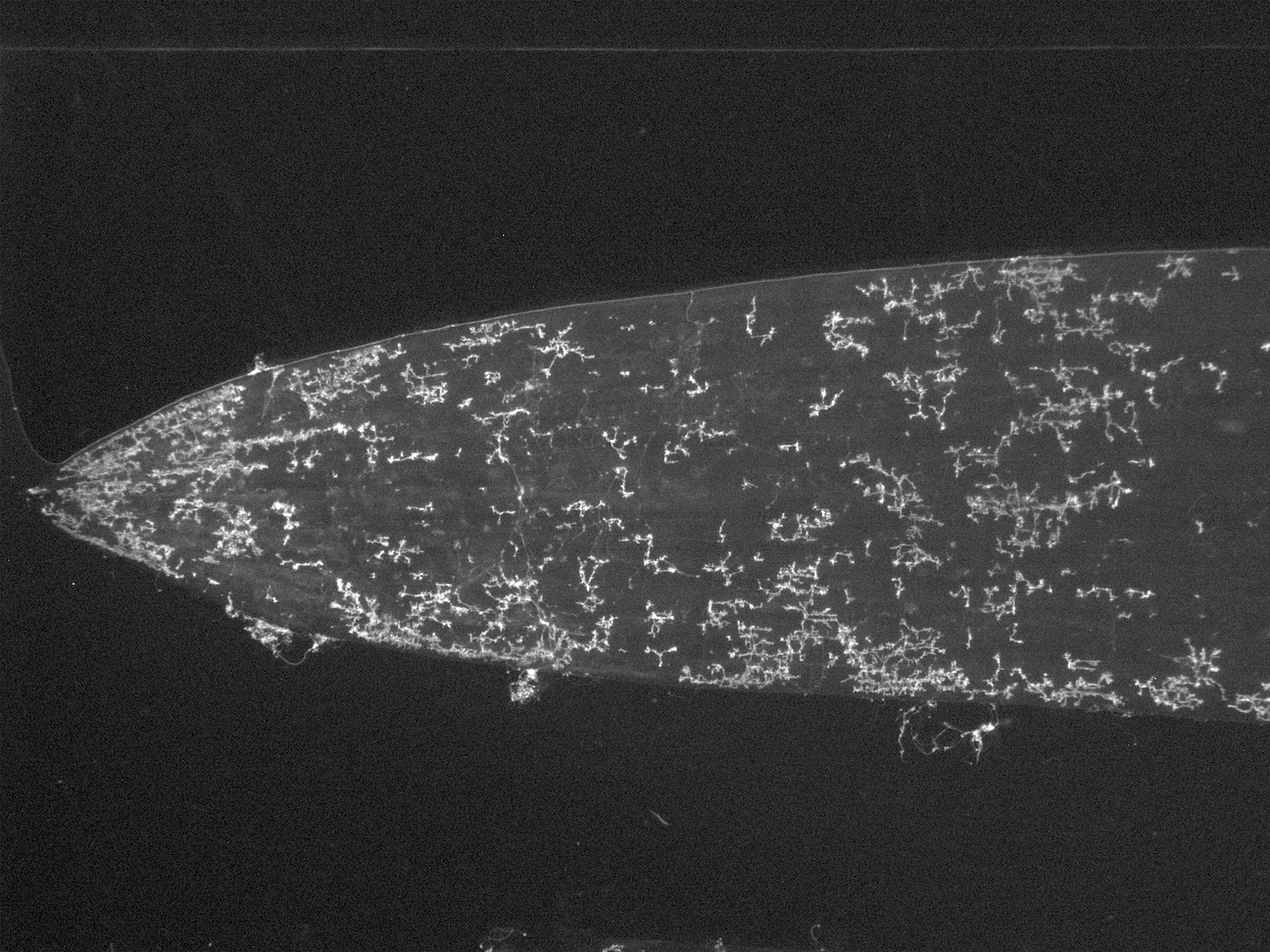

Supplement: Supplementary file 16 — Source Data [file 41467_2021_27288_MOESM16_ESM.zip › Source Data/Figure 1/micrographs/MM_687-348B_L_SxGP_DH-113.tif]

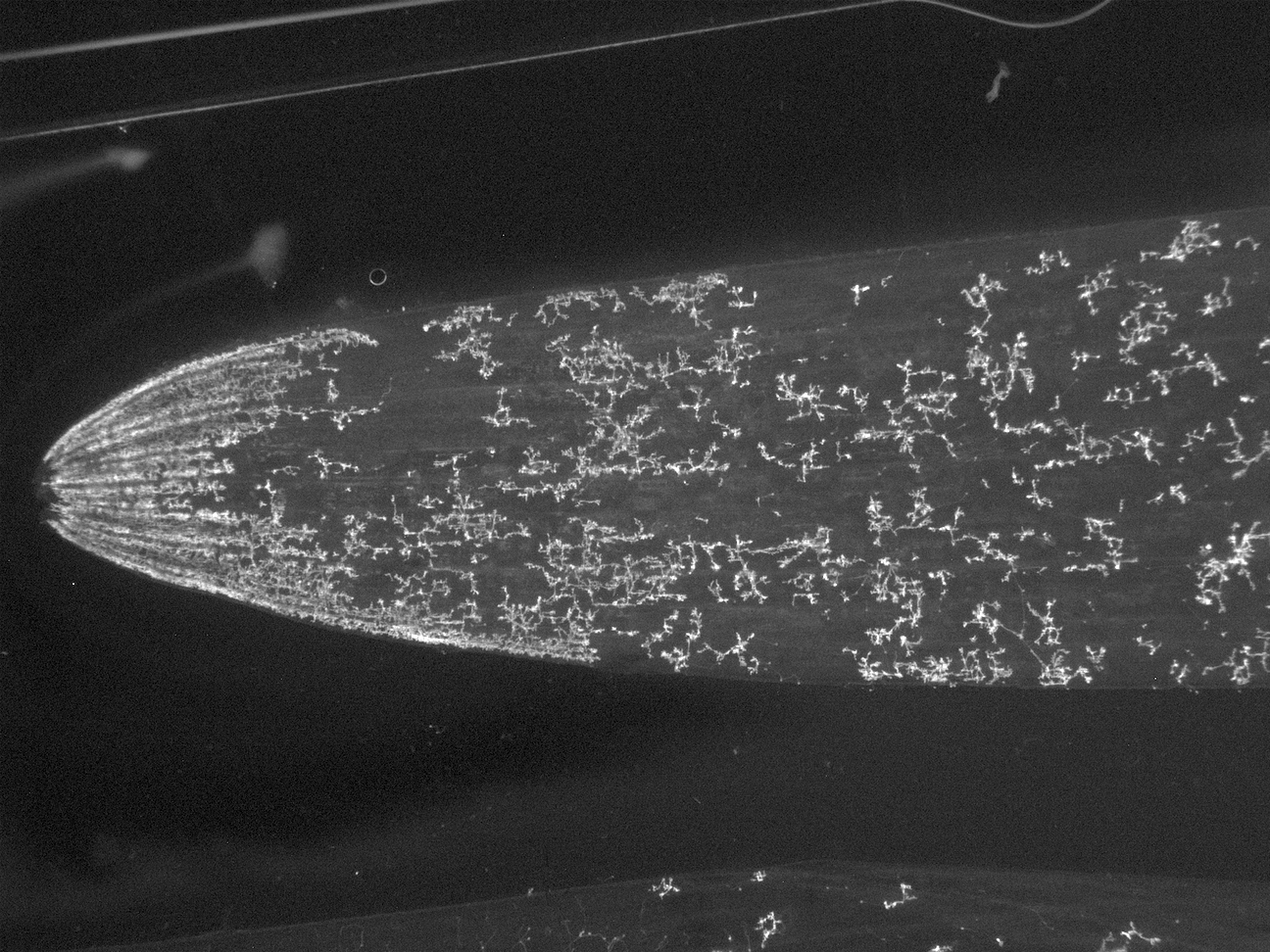

Supplement: Supplementary file 16 — Source Data [file 41467_2021_27288_MOESM16_ESM.zip › Source Data/Figure 1/micrographs/MM_687-236C_L_SxGP_DH-1.tif]

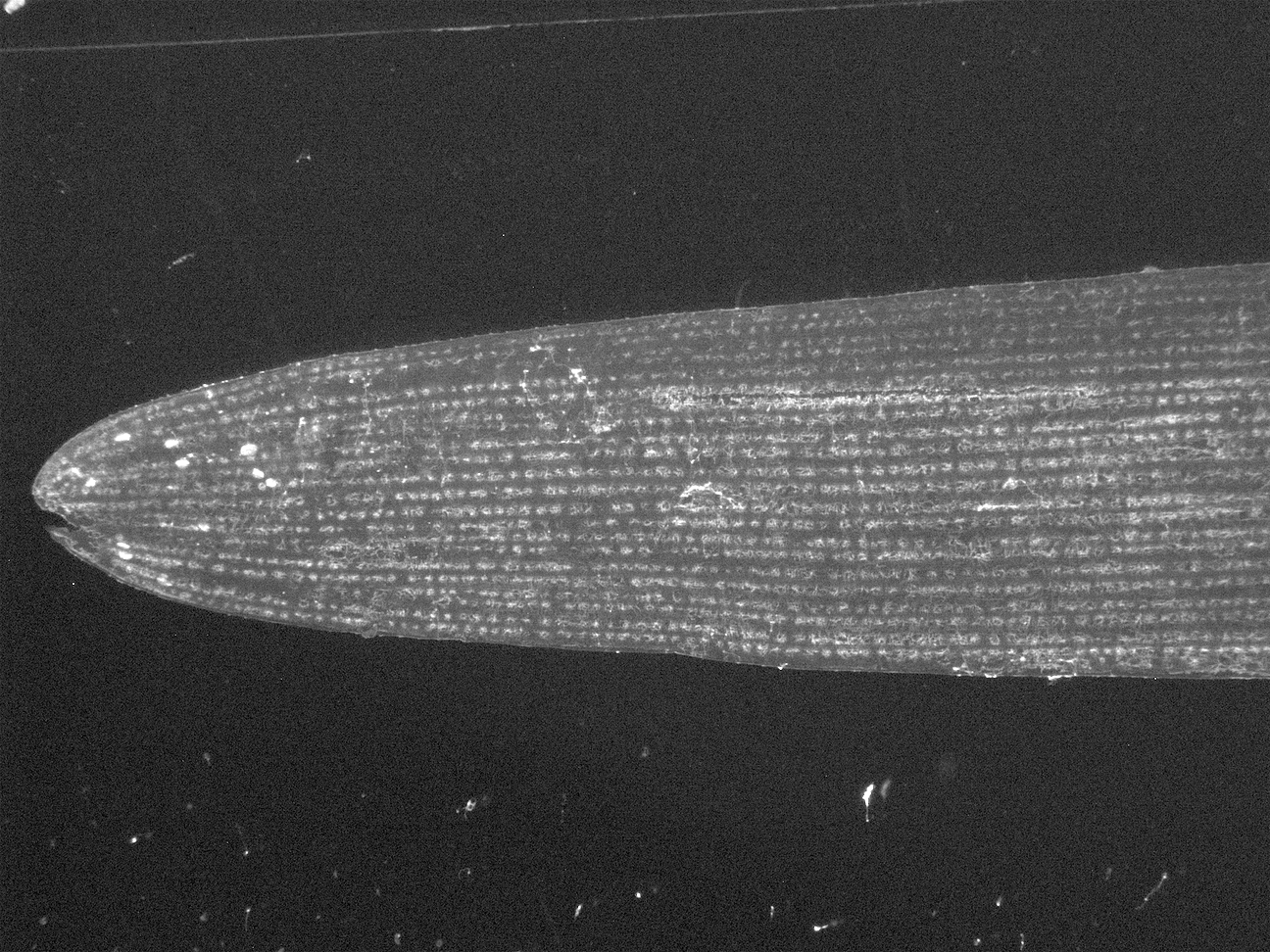

Supplement: Supplementary file 16 — Source Data [file 41467_2021_27288_MOESM16_ESM.zip › Source Data/Figure 1/micrographs/MM_687-314A_L_SxGP_DH-79.tif]

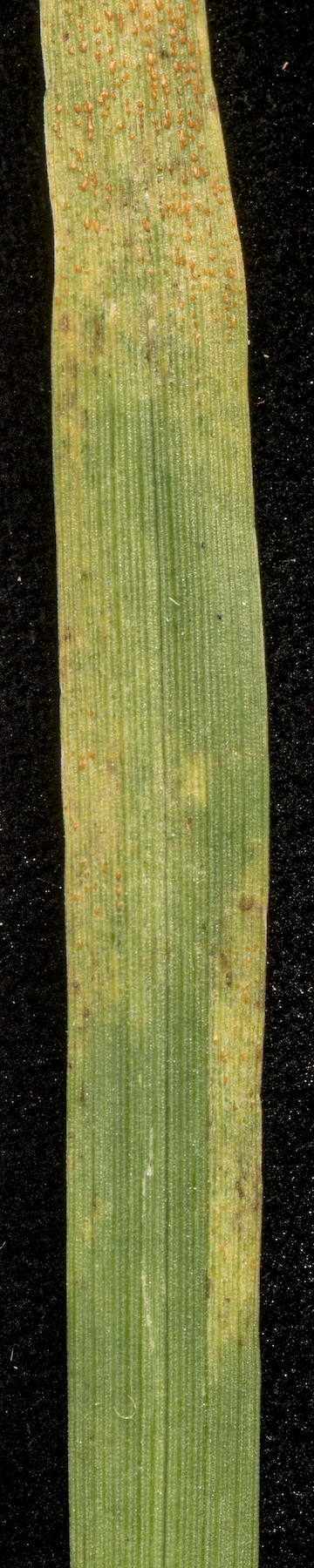

Supplement: Supplementary file 16 — Source Data [file 41467_2021_27288_MOESM16_ESM.zip › Source Data/Figure 5/cropped_images/_DSC0844_HVT_00227_2_3_4_Mla8_4.tif]

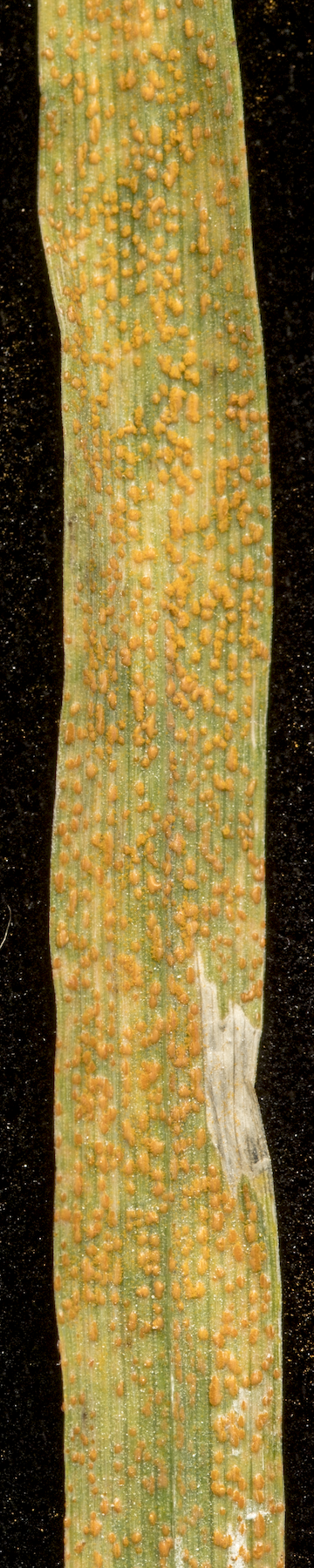

Supplement: Supplementary file 16 — Source Data [file 41467_2021_27288_MOESM16_ESM.zip › Source Data/Figure 5/cropped_images/_DSC0845_HVT_00250_1_2_3_Mla8_1.tif]

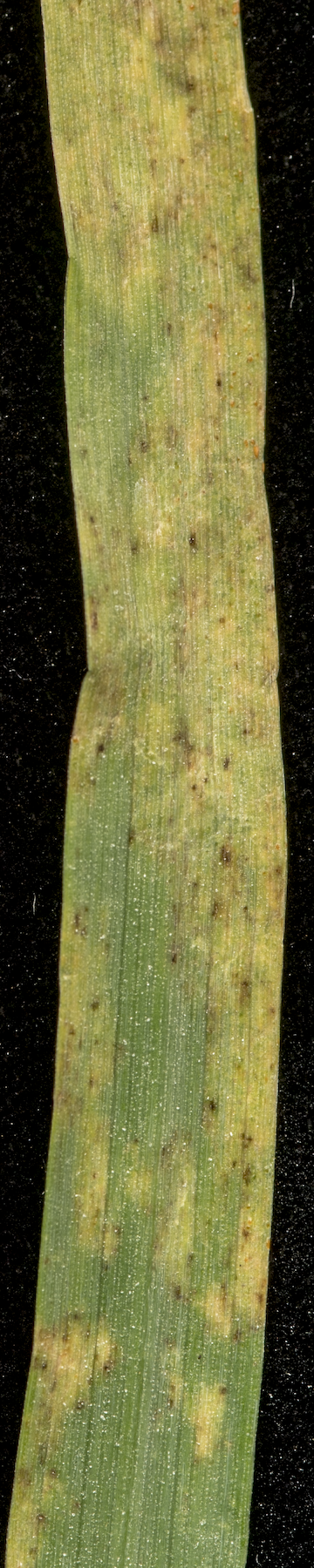

Supplement: Supplementary file 16 — Source Data [file 41467_2021_27288_MOESM16_ESM.zip › Source Data/Figure 5/cropped_images/_DSC0843_HVT_00215_1_2_3_Mla8_1.tif]

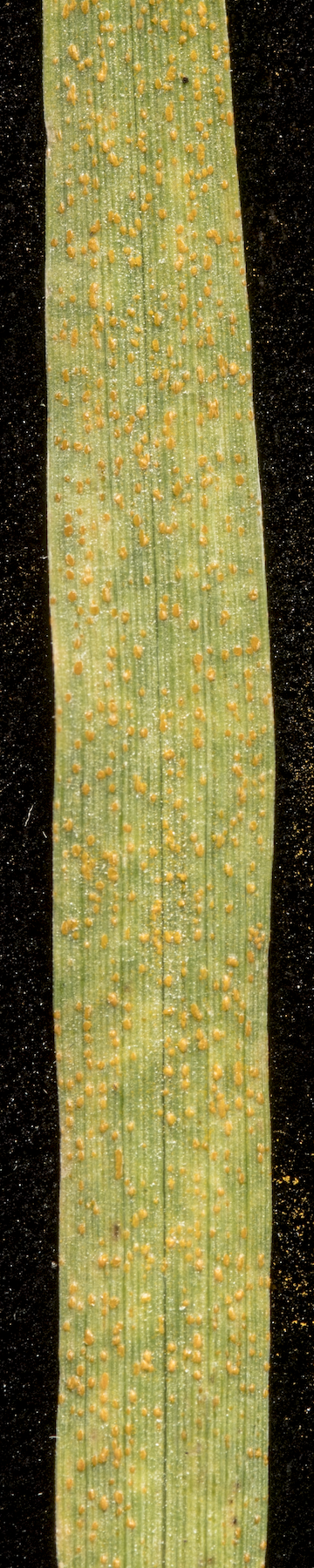

Supplement: Supplementary file 16 — Source Data [file 41467_2021_27288_MOESM16_ESM.zip › Source Data/Figure 5/cropped_images/_DSC0850_HVT_00261_2_3_4_Mla6_3.tif]

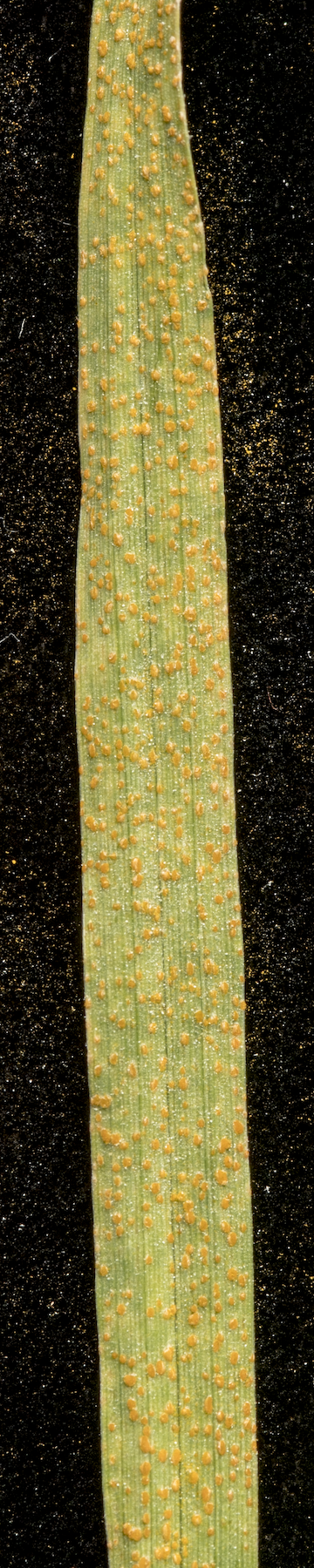

Supplement: Supplementary file 16 — Source Data [file 41467_2021_27288_MOESM16_ESM.zip › Source Data/Figure 5/cropped_images/_DSC0849_HVT_00388_1_2_3_Mla1_2.tif]

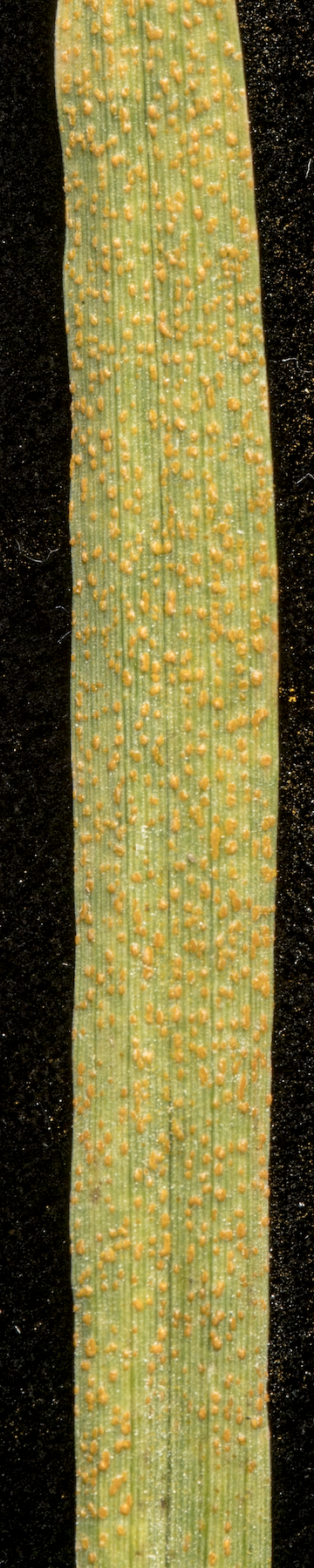

Supplement: Supplementary file 16 — Source Data [file 41467_2021_27288_MOESM16_ESM.zip › Source Data/Figure 5/cropped_images/_DSC0849_HVT_00388_1_2_3_Mla1_3.tif]

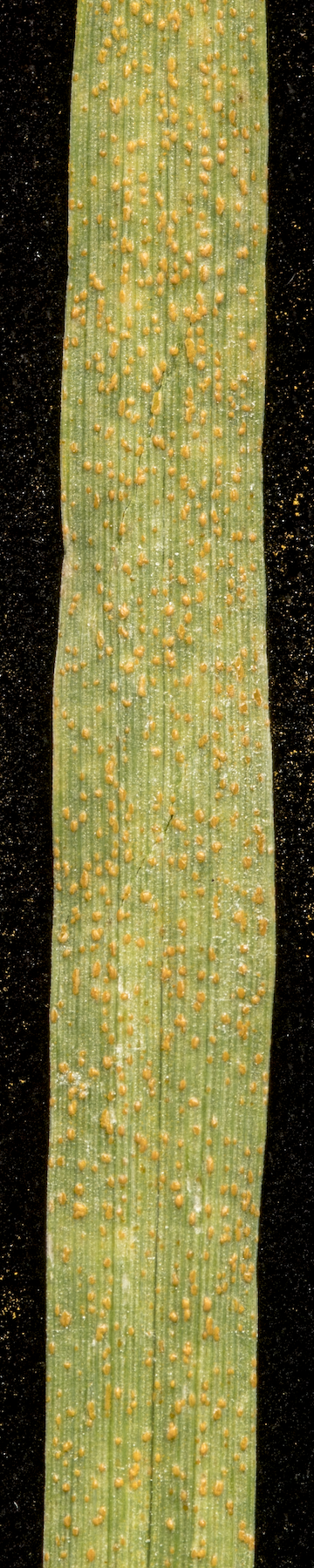

Supplement: Supplementary file 16 — Source Data [file 41467_2021_27288_MOESM16_ESM.zip › Source Data/Figure 5/cropped_images/_DSC0850_HVT_00261_2_3_4_Mla6_2.tif]

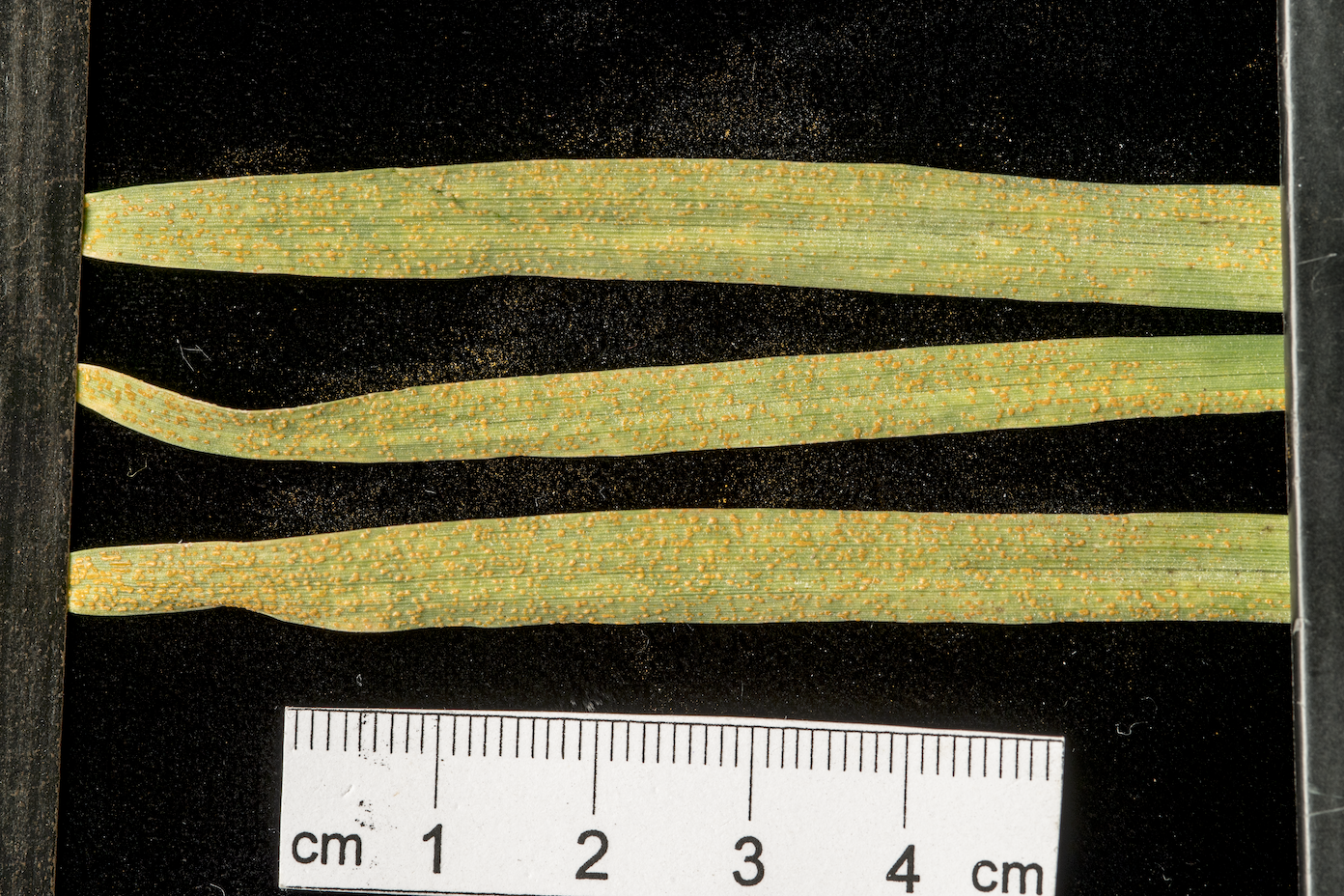

Supplement: Supplementary file 16 — Source Data [file 41467_2021_27288_MOESM16_ESM.zip › Source Data/Figure 5/raw_images/_DSC0849_HVT_00388_1_2_3_Mla1.tif]

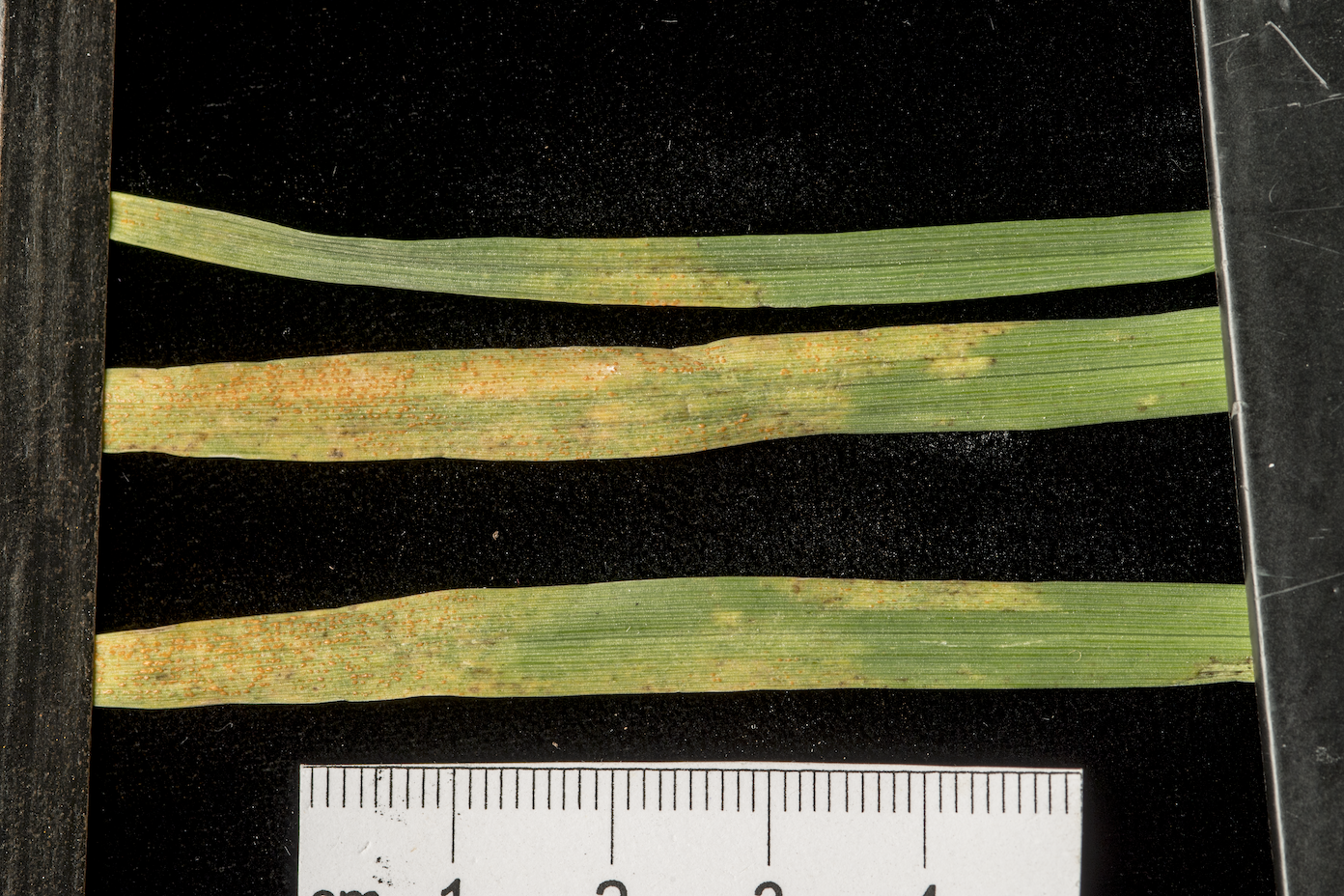

Supplement: Supplementary file 16 — Source Data [file 41467_2021_27288_MOESM16_ESM.zip › Source Data/Figure 5/raw_images/_DSC0844_HVT_00227_2_3_4_Mla8.tif]

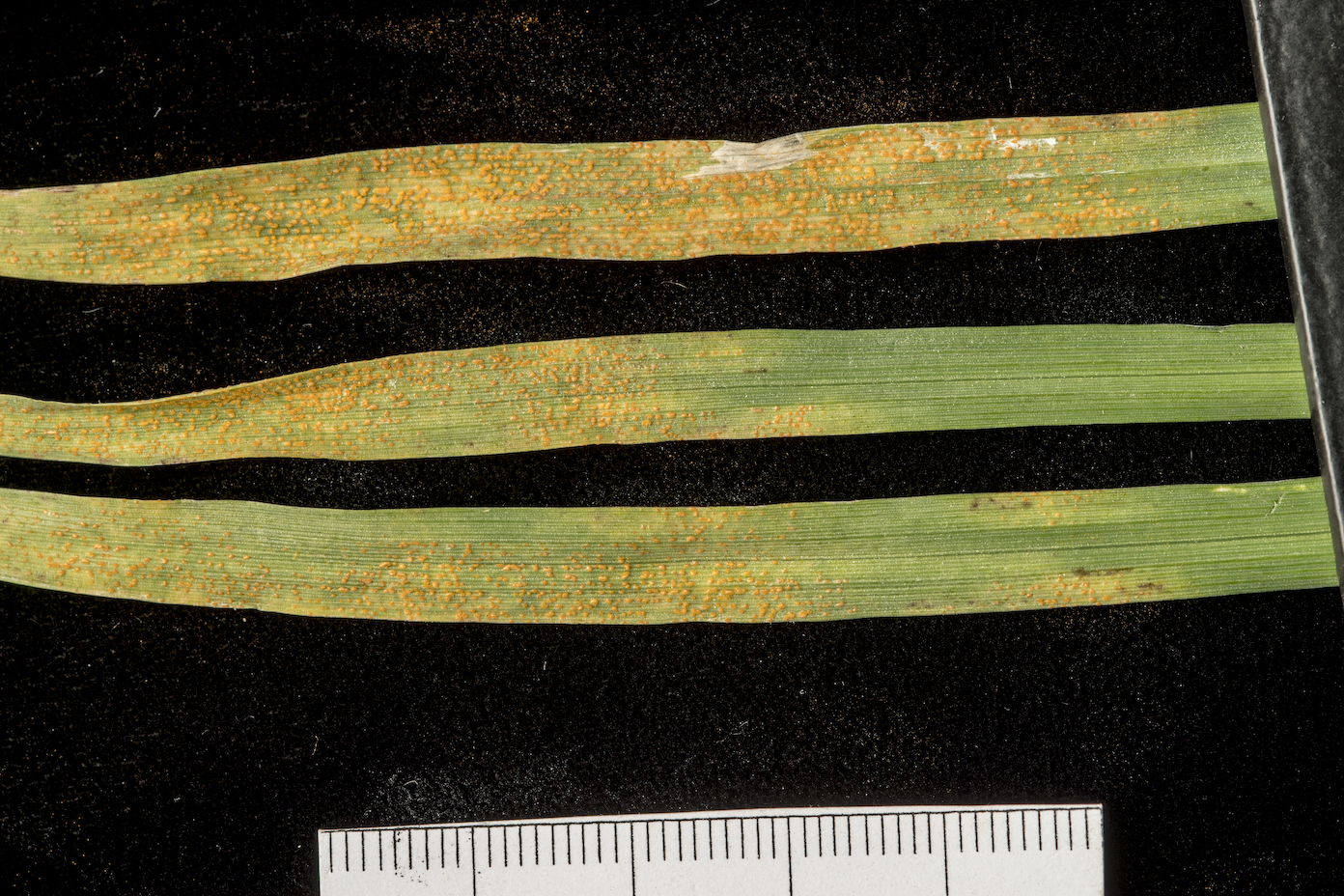

Supplement: Supplementary file 16 — Source Data [file 41467_2021_27288_MOESM16_ESM.zip › Source Data/Figure 5/raw_images/_DSC0845_HVT_00250_1_2_3_Mla8.tif]

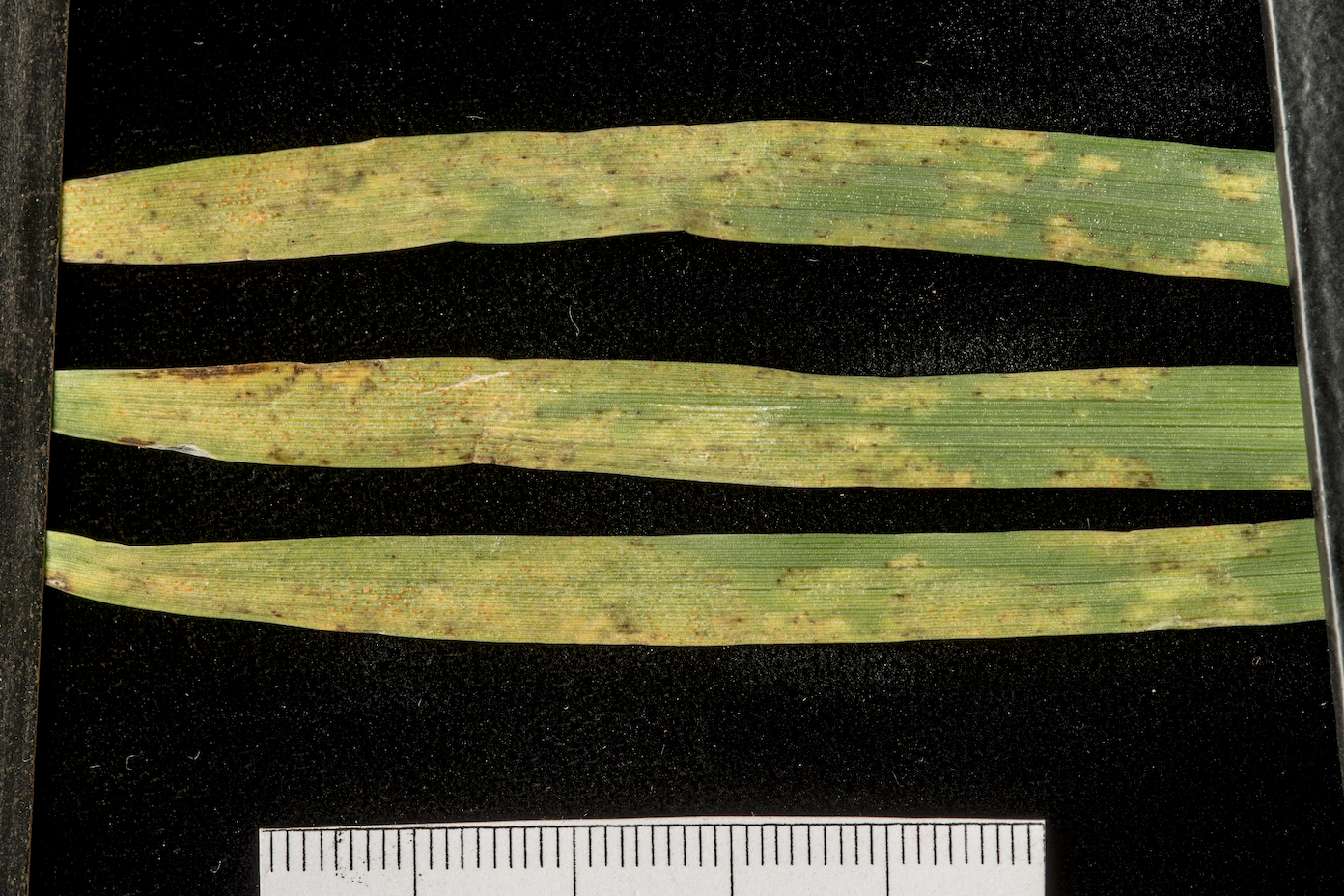

Supplement: Supplementary file 16 — Source Data [file 41467_2021_27288_MOESM16_ESM.zip › Source Data/Figure 5/raw_images/_DSC0843_HVT_00215_1_2_3_Mla8.tif]

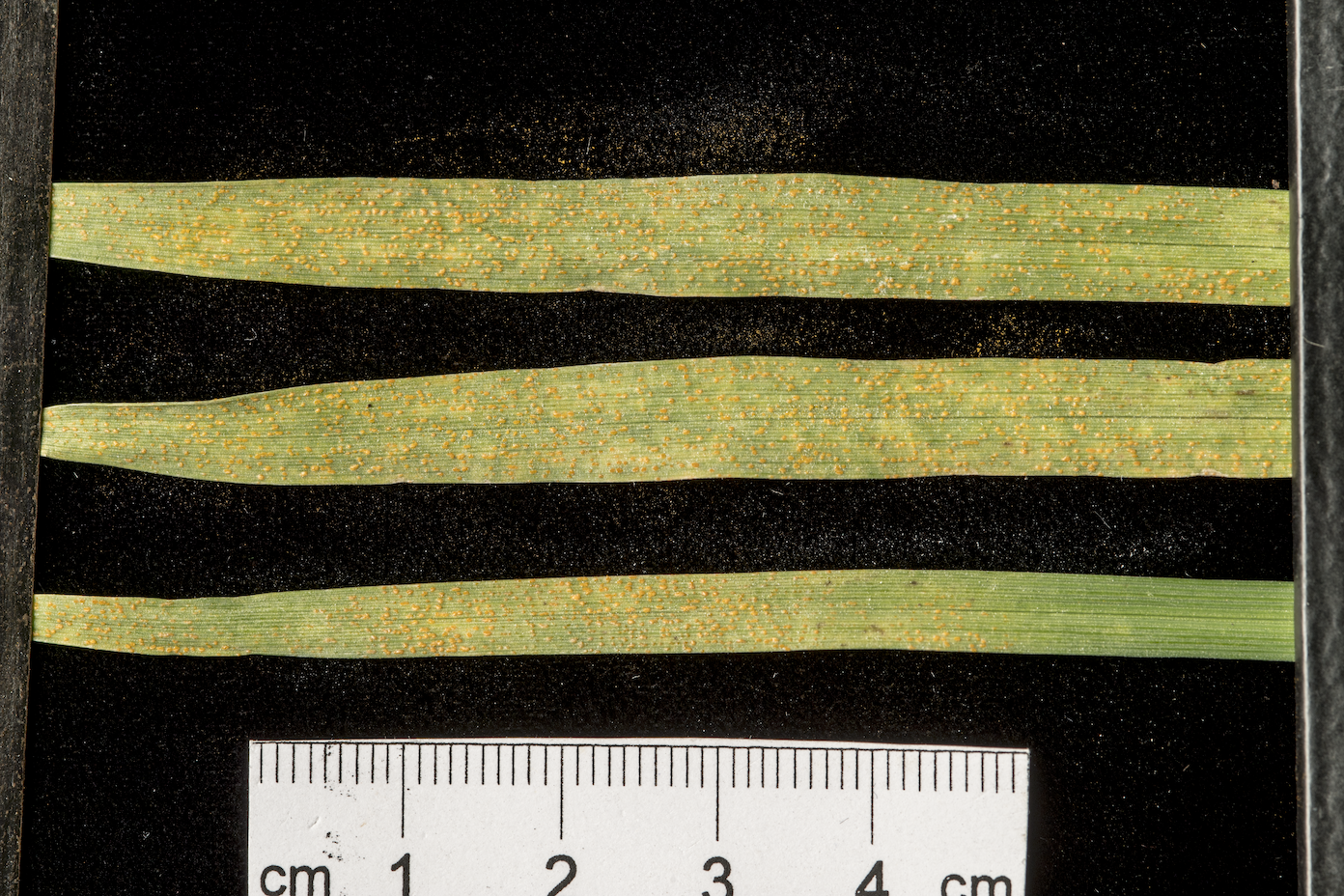

Supplement: Supplementary file 16 — Source Data [file 41467_2021_27288_MOESM16_ESM.zip › Source Data/Figure 5/raw_images/_DSC0850_HVT_00261_2_3_4_Mla6.tif]
